# Cidofovir selectivity is based on the different response of normal and cancer cells to DNA damage

**DOI:** 10.1186/1755-8794-6-18

**Published:** 2013-05-23

**Authors:** Tim De Schutter, Graciela Andrei, Dimitri Topalis, Lieve Naesens, Robert Snoeck

**Affiliations:** 1 Rega Institute for Medical Research, Laboratory of Virology and Chemotherapy, KU Leuven, Leuven, Belgium

**Keywords:** Cervical carcinoma, Cidofovir, DNA damage and repair, Gene expression profiling, Human papillomavirus

## Abstract

**Background:**

Cidofovir (CDV) proved efficacious in treatment of human papillomaviruses (HPVs) hyperplasias. Antiproliferative effects of CDV have been associated with apoptosis induction, S-phase accumulation, and increased levels of tumor suppressor proteins. However, the molecular mechanisms for the selectivity and antitumor activity of CDV against HPV-transformed cells remain unexplained.

**Methods:**

We evaluated CDV drug metabolism and incorporation into cellular DNA, in addition to whole genome gene expression profiling by means of microarrays in two HPV^+^ cervical carcinoma cells, HPV^-^ immortalized keratinocytes, and normal keratinocytes.

**Results:**

Determination of the metabolism and drug incorporation of CDV into genomic DNA demonstrated a higher rate of drug incorporation in HPV^+^ tumor cells and immortalized keratinocytes compared to normal keratinocytes. Gene expression profiling clearly showed distinct and specific drug effects in the cell types investigated. Although an effect on inflammatory response was seen in all cell types, different pathways were identified in normal keratinocytes compared to immortalized keratinocytes and HPV^+^ tumor cells. Notably, Rho GTPase pathways, LXR/RXR pathways, and acute phase response signaling were exclusively activated in immortalized cells. CDV exposed normal keratinocytes displayed activated cell cycle regulation upon DNA damage signaling to allow DNA repair via homologous recombination, resulting in genomic stability and survival. Although CDV induced cell cycle arrest in HPV^-^ immortalized cells, DNA repair was not activated in these cells. In contrast, HPV^+^ cells lacked cell cycle regulation, leading to genomic instability and eventually apoptosis.

**Conclusions:**

Taken together, our data provide novel insights into the mechanism of action of CDV and its selectivity for HPV-transformed cells. The proposed mechanism suggests that this selectivity is based on the inability of HPV^+^ cells to respond to DNA damage, rather than on a direct anti-HPV effect. Since cell cycle control is deregulated by the viral oncoproteins E6 and E7 in HPV^+^ cells, these cells are more susceptible to DNA damage than normal keratinocytes. Our findings underline the therapeutic potential of CDV for HPV-associated malignancies as well as other neoplasias.

## Background

Human papillomaviruses (HPVs) are small double-stranded DNA viruses with a strict epithelial tropism. HPVs infect either mucosal or cutaneous surfaces causing a variety of diseases ranging from benign warts (low-risk types) to malignant neoplasms, including cervical carcinoma and other anogenital cancers (high-risk types) [1]. The virus infects cells in the basal layer of stratified squamous epithelia and viral replication shows both temporal and spatial regulation of viral protein expression. Except for E1 (replicative helicase) and E2 (DNA-binding regulatory protein), HPV completely relies on the cellular DNA synthesis machinery for its genome replication [2]. Development of HPV-induced cancerous lesions is often accompanied by partial integration of the viral genome in the host cell DNA, resulting in conservation and stabilized expression of E6 and E7 oncoproteins [3]. Other parts of the viral genome are usually either deleted or show a disturbed expression [4]. Therefore, cell lines derived from cervical carcinomas do not produce HPV virions and only express the E6 and E7 oncoproteins [5,6].

These two viral oncogenes cooperate in cell transformation and immortalization [7]. The E7 oncoprotein overrides the G1/S checkpoint of the cell cycle through association with the retinoblastoma family of proteins (pRb, p107 and p130). Via induction of their ubiquitin-mediated proteolysis, and disruption of their association with the E2f family of transcription factors, E7 activates expression of several S-phase specific genes [8]. E7 also alters cell cycle control through interactions with histone deacetylases, cyclins and cyclin-dependent kinase inhibitors (p21^CIP1^ and p27^KIP1^) that are important regulators of growth arrest during epithelial differentiation [7]. As a result of pRb degradation, other activities of this tumor suppressor protein, such as DNA repair and maintenance of genomic integrity, are also abrogated. E7 expression causes stabilization and functional impairment of the tumor suppressor protein p53 resulting in stimulation of apoptosis. To counteract this, E6 proteins target p53, leading to ubiquitinylation and proteasomal degradation of p53, preventing cell growth arrest and apoptosis [9]. E6 proteins also activate telomerase expression and regulate the activities of PDZ domain-containing proteins and tumor necrosis factor receptors. Both E6 and E7 induce genomic instability and also target cytokine expression to control cell proliferation and interferon responses [7].

HPV-related malignancies, other than cervical cancer, have increased in the last years because of the higher number of immunocompromised patients. Current treatment modalities for HPV-associated anogenital hyperplasia rely on removal of the lesions and are often mutilating, painful and associated with high recurrence rates. New medical therapies, such as intralesional or topical administration of cidofovir (CDV, Vistide®), which maintain the anatomical integrity and sexual function of the patients need to be further investigated. Cidofovir, approved by the FDA for intravenous administration in the therapy of cytomegalovirus retinitis in AIDS patients, has a broad-spectrum anti-DNA virus activity, including HPVs [10]. Its antiviral activity against viruses that encode for their own DNA polymerases (herpes-, pox-, and adenoviruses) is based on a higher affinity of the active diphosphate metabolite (CDVpp) for viral DNA polymerases compared to cellular DNA polymerases [10].

CDV can be used intravenously, intralesionally or topically. Systemic administration requires co-administration of oral probenecid and intravenous hydration to prevent nephrotoxicity. Topical cidofovir is a simple and usually well-tolerated therapy with minimal, if any, side effects (ulcerations at the site of affected mucosa but not on the surrounding normal tissue). These local side effects, when appearing, are self-healing and do not require cessation of treatment. Despite the fact that HPVs do not encode for their own DNA polymerase, off-label use of cidofovir was effective in the treatment of high-risk HPV-associated hyperplasia’s including, cervical [11,12], vulvar [13-15], perianal [14], gingival and buccal [16], and hypopharyngeal and esophageal [17] neoplasias.

*In vitro*, CDV has been shown to exert antiproliferative effects against HPV-positive (HPV^+^) cervical carcinoma cells, and to a lower extent against HPV-negative (HPV^-^) immortalized cells [18]. The antiproliferative effect of CDV was ascribed to apoptosis induction, accumulation of cells in S-phase, and induction of p53, pRb and p21 protein expression [19,20]. A synergistic effect of CDV and radiation in HPV^+^ cervical carcinoma cells [21] and in head and neck squamous cell carcinoma cells [22] was associated with p53 accumulation. The stromal-derived factor 1 (SDF-1α)-stimulated invasiveness of HPV^+^ cells was abrogated by CDV and this anti-metastatic action was mediated by inhibition of E6/E7, CXCR4 and Rho/ROCK signaling [23]. To explain the selectivity of CDV for HPV-transformed cells, it was suggested that CDV could be differentially metabolized in HPV16^+^ cells *versus* human keratinocytes [24]. However, the molecular mechanisms underlying the selectivity of CDV for HPV remain unexplained.

Gene expression profiling has proven successful in identifying the mechanism of action of pharmaceutical agents [25,26]. In this study, we evaluated gene expression changes following CDV treatment of different cell types [including, two HPV^+^ cervical carcinoma cell lines (SiHa and HeLa), an HPV^-^ immortalized keratinocyte cell line (HaCaT), and primary human keratinocytes (PHKs)] to provide more insights into the mode of action and selectivity of CDV. Furthermore, metabolic studies and drug incorporation into genomic DNA were analyzed in the four cell types.

## Methods

### Antiviral compound

Cidofovir (CDV), obtained from Gilead Sciences (Foster City, CA, USA), was prepared as 10 mg/ml solution in PBS. [5-^3^H]-CDV (1 mCi/ml; specific activity: 26 Ci/mmol) was synthesized by Moravek Biochemicals (Brea, CA, USA), and stored at −20°C in ethanol/water 1:1.

### Cell cultures

The following cell types were used: HPV16^+^ (SiHa) and HPV18^+^ (HeLa) cervical carcinoma cell lines, HPV^-^ human immortalized keratinocytes (HaCaT) and primary human keratinocytes (PHKs). SiHa, HeLa and HaCaT cells were maintained in Dulbecco’s modified Eagle’s medium (Gibco®, Life Technologies™, Invitrogen, Belgium) supplemented with 10% fetal calf serum. PHKs were isolated from neonatal foreskins as described previously [27] and cultured in Keratinocyte-SFM Medium (Gibco®, Life Technologies™).

### Total RNA extraction

Cells pellets containing 10^6^ cells were lysed with TRIzol reagent (Invitrogen, Belgium) for 3 minutes at room temperature. Chloroform, 20% of total volume, was added to the mixture which was subsequently centrifuged (13,000 rpm) at 4°C for 15 minutes. The upper aqueous layer containing the RNA was recovered and mixed with an equal volume of 70% ethanol. The RNA was further purified by RNeasy Mini Kit (Qiagen Benelux, Netherlands) according to manufacturer’s instructions. Concentration and purity of RNA was determined with a NanoDrop ND1000 device (Fisher Scientific, Belgium). Integrity of RNA samples was verified by standard denaturing agarose gel electrophoresis. For microarray experiments, RNA quality was also assessed by an Agilent Bioanalyzer system (Agilent, Belgium).

### Gene expression profiling by microarrays

Human Genome U133 Plus 2.0 arrays (Affymetrix, CA, USA) were used to analyze whole genome gene expression in a single hybridization, containing more than 54,000 probe sets and covering approximately 38,500 genes. Array hybridization, scanning and image analyzing were done according to the manufacturer’s protocols (Affymetrix GeneChip Expression Assay) at the VIB Nucleomics Core Facility (http://www.nucleomics.be).

Three different microarray experiments were carried out to evaluate gene expression changes following 50 μg/ml CDV treatment: experiment ‘1’ included a wide range of treatment periods (between 2 h and 120 h) of SiHa cells using one microarray per time point and per condition; experiment ‘2’ consisted of SiHa cells treated for 24 h, 48 h, and 72 h; experiment ‘3’ comprised HeLa, HaCaT, and PHK exposed to CDV for 72 h. In the second and third experiments, gene expression profiling was explored by triplicate testing.

### Analysis of microarray data

Raw data were corrected for background signal using the RMA (Robust Multi-array analysis) algorithm (affy_1.22.0 package of BioConductor) that normalizes the data so that different arrays can be compared to each other and summarizes the data into expression values [28]. The detection (Present/Absent) call generated by the Affymetrix microarray suite version 5 software (MAS 5.0) was used to remove probe sets that were not reliable detected in any of the microarrays before further analysis.

Differentially expressed (DE) probe sets between CDV treated and untreated cells were determined using a moderated t-statistic test [LIMMA (linear models for microarray data), BioConductor]. The Benjamini Hochberg correction for multiple testing [p < 0.05, false discovery rate (FDR) = 0.05] was performed [29]. Probe sets were considered significantly DE if the absolute fold change (FC) was > 2 and the *P*-value was < 0.05 (LIMMA) after applying the Benjamini-Hochberg correction. The resulting list of relative gene expression levels for a given condition was designated as a data set (Additional file 1: Figure S1).

### Microarray data accession number

The entire set of microarray data is deposited in the Gene Expression Omnibus (GEO, http://www.ncbi.nlm.nih.gov/projects/geo) according to MIAME standards under accession numbers GSE26748 (SiHa data) and GSE39293 (HeLa, HaCaT, and PHKs data), respectively:

http://www.ncbi.nlm.nih.gov/geo/query/acc.cgi?token=lpivfquymowyazo&acc=GSE26748

http://www.ncbi.nlm.nih.gov/geo/query/acc.cgi?token=lbqtpommkiccudo&acc=GSE39293

### Bioinformatics analysis of differentially expressed (DE) genes

Ingenuity Pathways Analysis (IPA, Ingenuity® Systems, Redwood City, CA, USA; http://www.ingenuity.com) version 9 was used to perform functional, transcription factor, and canonical pathway analysis. The IPA application reveals relevant pathways and biological functions by comparing the number of genes that participate in a given function or pathway, relative to the total number of occurrences of those genes in all the pathways stored in the IPKB (Ingenuity Pathway Knowledge Base).

Data sets with the corresponding FC and *P*-value were uploaded into the IPA software. Stringent criteria, equivalent to those described for the selection of DE probes, were applied to identify DE genes. When genes were represented by 2 or more probe sets on the arrays, only the maximum FC was used. Uncharacterized probe sets were not included in the analysis. Networks were built by determining all interactions among genes categorized with the functional analysis.

### RT-PCR analysis

To validate the microarray data, expression levels of selected genes were determined by real-time RT-PCR using the TaqMan® Fast Universal PCR Master Mix and TaqMan® Gene Expression Assays from Applied Biosystems (Foster City, CA, USA). Equal amounts of total RNA isolated from CDV treated and untreated cells were transcribed to cDNA with the First-Strand cDNA Synthesis Kit (GE Heathcare, Little Chalfont, UK) following manufacturer’s instructions. RT-PCR was performed on a 7500 Fast Real-Time PCR System (Applied Biosystems, Foster City, CA, USA) according to manufacturer’s instructions. Relative expression levels were calculated with the ΔΔC_T_ method, using β-actin as endogenous control (Applied Biosystems).

The expression of the two HPV16 oncogenes E6 and E7 in SiHa cells was also quantified with RT-PCR. The cDNA’s were prepared as described above and RT-PCR was also carried out under the same experimental conditions. The following forward (F) and reverse (R) primers and probes (P) were used: HPV16_E6_F: 5′-AGAACTGCAATGTTTCAGGACC-3′, HPV16_E6_R: 5′-TGTATAGTTGTTTGCAGCTCTGTGC-3′, HPV16_E6_P: (FAM) 5′-ACAGGAGCGACCCAGA-3′, HPV16_E7_F: 5′-GCTCAGAGGAGGAGGATGAAATAGA-3′, HPV16_E7_R: 5′-GAGTCACACTTGCAACAAAAGGTT-3′, HPV16_E7_P: (FAM) 5′-TCCGGTTCTGCTTGTCC-3′.

### Metabolism study with [5-^3^H]-CDV

Radioactive [5-^3^H]-labeled CDV was used to evaluate the metabolism in the different cell types. Cells (2 × 10^6^ cells per 75 cm^2^ flask) were incubated with [5-^3^H]-CDV at a final concentration of 50 μg/ml and 10 μCi per flask. After 72 h incubation at 37°C, samples for HPLC analysis were prepared by methanol extraction as described previously [30]. For HPLC analysis, 200 μL extract was injected onto an anion-exchange Partisphere SAX column (dimensions: 4.6 mm × 125 mm) from Whatman (Maidstone, UK), and separated with two phosphate buffers (A: 5 mM and B: 0.3 M ammonium dihydrogen phosphate; both at pH 3.5), and the following gradient (flow: 2 ml/min): 100% A (5 min); linear gradient to 100% B (15 min); 100% B (20 min); linear gradient to 100% A (5 min) and 100% A (15 min). One minute fractions of the eluate were collected, mixed with Hisafe 3 cocktail (Perkin Elmer, Waltham, MA) and analyzed for radioactivity in a scintillation counter. The retention times of the different CDV metabolites were: 3 min for CDVp-choline; 5 min for CDV; 15 min for CDVp; and 19 min for CDVpp.

To determine incorporation of [5-^3^H]-CDV into cellular nucleic acid material, the methanol-insoluble pellets were digested in 500 μl 5 M sodium hydroxide during 24 h incubation at 37°C. Sodium hydroxide extracts were neutralized with 500 μl 5 M hydrochloride. Nucleic acid samples were transferred to scintillation vials, mixed with Hisafe 3 cocktail (Perkin Elmer, Waltham, MA) and analyzed for total radioactivity in a scintillation counter. All conditions were performed in duplicate.

## Results

### Metabolism and incorporation

Since CDV has been suggested to be preferentially converted to its active diphosphate form (CDVpp) in HPV16^+^ cells [24], we investigated the metabolism of [5-^3^H]-CDV in HPV^+^ cervical carcinoma cells compared to HPV^-^ immortalized keratinocytes and normal keratinocytes. Following 72 h incubation with the compound, CDV-phosphocholine (which is considered the intracellular depot form of CDV) appeared to be the most abundant metabolite while the monophosphate form (CDVp) was the least abundant one in all four cell types. No significant differences in the levels of the active metabolite (CDVpp), CDV-phosphocholine or CDV were observed between PHKs and HPV^+^ tumor cells. However, lower CDVp levels were measured in PHKs compared to HPV^+^ cells following 72 h incubation.

Notably, lower concentrations of CDV and of all metabolites were observed in HaCaT cells, compared to either HPV^+^ cells or PHKs (Table 1), suggesting that HaCaT cells have a different uptake and/or efflux of CDV, rather than differences in drug metabolism.

**Table 1:** CDV metabolism and incorporation

	**SiHa [pmol/10e^6^ cells]**	**HeLa [pmol/10e^6^ cells]**	**HaCaT [pmol/10e^6^ cells]**	**PHKs [pmol/10e^6^ cells]**
*Methanol-soluble fraction*				
CDV	4.55 ± 0.03	3.20 ± 0.25	1.76 ± 0.11	4.62 ± 0.63
CDVp	2.35 ± 0.13	1.61 ± 0.04	0.38 ± 0.08	0.78 ± 0.14
CDVpp	3.50 ± 0.02	2.93 ± 0.08	1.56 ± 0.01	3.56 ± 0.28
CDVp-choline	11.90 ± 0.37	8.73 ± 0.24	2.65 ± 0.12	9.18 ± 1.34
Sum of all metabolites	22.29 ± 0.23	16.47 ± 0.06	6.34 ± 0.08	18.13 ± 2.39
*Digestion of methanol- insoluble fraction*				
Incorporated CDV	18.39 ± 0.54	13.17 ± 1.43	8.62 ± 1.11	1.96 ± 0.71

Cells were incubated for 72 h with 10 μCi radiolabeled CDV (final concentration of 50 μg/ml) to asses metabolism and incorporation in cellular DNA. Data are the mean ± SD of two experiments.

To compare the efficiency of CDV incorporation into genomic DNA in the different cell types, we performed an analysis of the methanol-insoluble pellets obtained from each cell type after incubation with radiolabeled compound for 72 h. Although the levels of intracellular CDV metabolites were not strikingly different in PHKs compared to immortalized keratinocytes and HPV^+^ tumor cells, evaluation of the methanol-insoluble fractions revealed important differences between the distinct cell types with higher amounts of CDV incorporated in tumor cells compared to normal keratinocytes. Following 72 h incubation, 2 pmol of CDV was found in the methanol-insoluble fraction per million cells for PHKs while at least 4-fold (HaCaT), 6-fold (HeLa) and 9-fold (SiHa) higher levels were determined in the immortalized keratinocytes and HPV^+^ tumor cells (Table 1).

These data indicate that CDVpp is more effective in terms of inhibition of cellular DNA synthesis leading to S-phase arrest for malignant cells than for normal cells. The higher incorporation of CDV into cellular DNA observed in HPV^+^ malignant cells compared to normal cells is in agreement with the selectivity of this compound for tumor cells. To investigate the consequences of this differential incorporation of CDV into cellular DNA, whole human genome gene expression profiling was performed.

### Gene expression profiling

#### Kinetic study of gene expression changes

**Figure 1:**
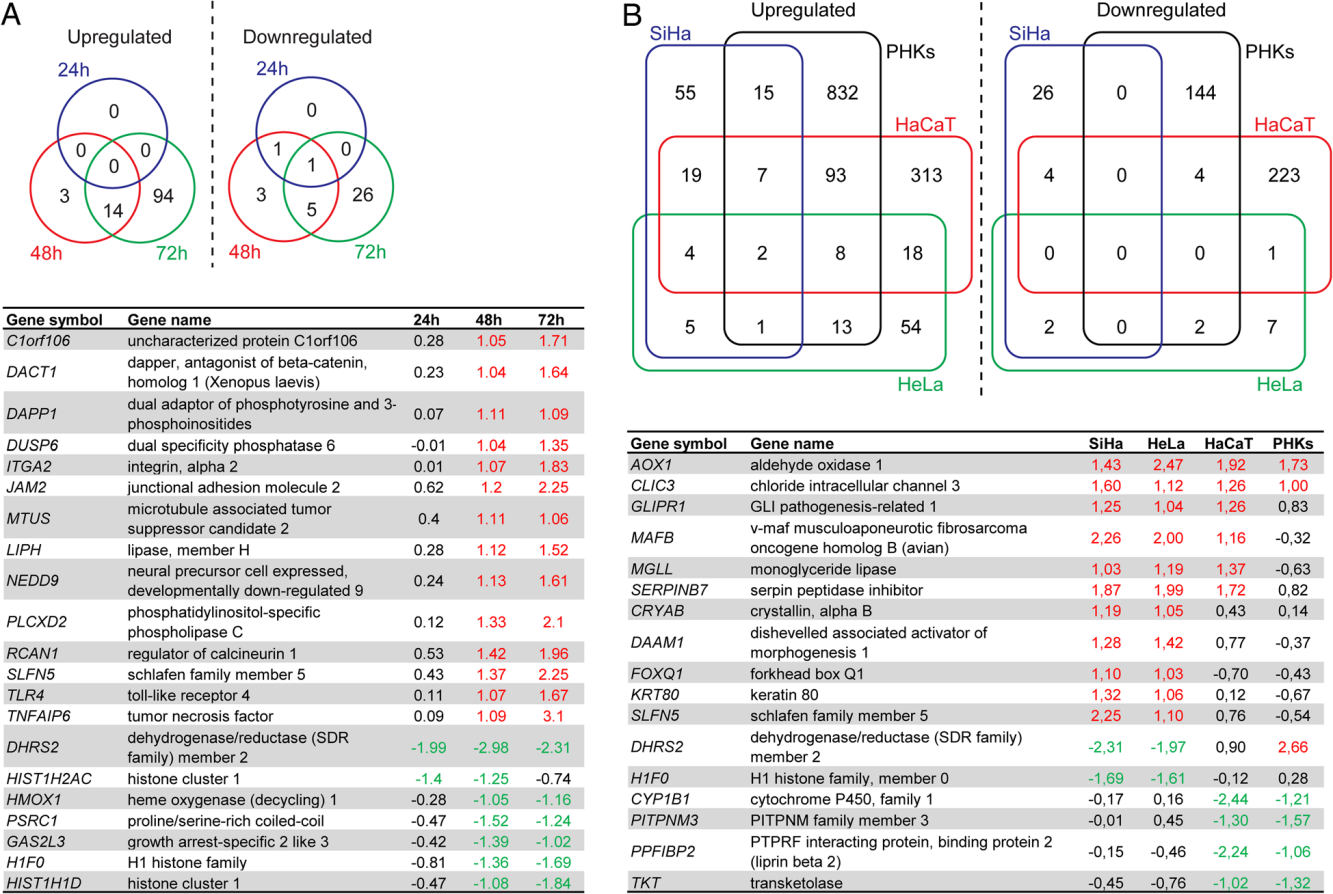
**Venn diagram representation of differentially expressed genes in the microarray experiments.** (**A**) Venn diagram analysis identifying the common and exclusively DE genes in SiHa cells treated with CDV for 24 h, 48 h, and 72 h and table listing the genes that were DE (log_2_ fold) at 24 h or 48 and 72 h post-treatment. (**B**) Venn diagram analysis of DE genes among the four cell types following treatment with CDV for 72 h and table listing the genes that were only up- or downregulated (log_2_ fold) in all four cell types, only in HPV^+^ cells, or only in HPV^-^ cells.

First, a kinetic study was performed to assess gene expression changes in SiHa cells incubated in the presence or absence of CDV for different times (from 2 h to 120 h). Considering the minimal changes observed up till 24 h following CDV addition, a second kinetic was performed that included treatment for 24 h, 48 h and 72 h. After 24 h, only 2 genes (*DHRS2* and *HIST1H2AC*) were downregulated, while no genes were found to be upregulated. Venn diagrams (Figure 1A) were used to classify the total number of genes whose expression change was specific to or common in the comparisons of CDV treatment for 24 h, 48 h and 72 h. The number of differentially expressed (DE) genes increased with the duration of CDV exposure. A total of 27 and 140 genes were DE after, respectively, 48 h and 72 h of CDV administration, the majority of the genes being upregulated (17 and 108 genes after 48 h and 72 h, respectively). Out of the 27 genes that showed an altered expression level following 48 h of treatment with CDV, 20 showed a similar alteration after 72 h (Figure 1A).

#### Comparison of gene expression profiling among different cell types

Based on the kinetic study and taking into account the overlap between the 48 h and 72 h data, the impact of CDV on gene expression in different cell types was evaluated at 72 h post-administration of the compound. To investigate the selectivity of CDV for HPV^+^ tumor cells and whether the presence of HPV affects the response to CDV, an HPV18^+^ carcinoma cell line (HeLa), an HPV^-^ immortalized keratinocyte cell line (HaCaT), and normal keratinocytes (PHKs) were evaluated in addition to SiHa cells (Additional file 1: Figure S1).

A comparison of the total number of genes that were found to be DE among the four cell types is depicted with Venn diagrams (Figure 1B). Similarly to SiHa cells, most of the DE genes were upregulated in HeLa, HaCaT and PHKs. The number of genes with deregulated expression was higher in HPV^-^ than in HPV^+^ cell types. The vast majority of DE genes following CDV incubation did not overlap between the different cell types. Only two genes (*AOX1* and *CLIC3*) were upregulated in all four tested cell types. Genes with reduced expression levels common to all four cell types were not detected (Figure 1B).

Different types of analysis (functional, upstream regulator, and pathway analysis) were performed with the four microarray data sets through the use of Ingenuity Pathways Analysis (IPA, Ingenuity® Systems). A comparison of the functional annotations upregulated or downregulated following CDV treatment in the four cell types is shown in Additional file 2: Figure S2 and a complete list with all identified canonical pathways affected by CDV is given in Additional file 3: Table S1. The upstream regulator analysis, a novel approach to transcription factor prediction, was used to predict activation or inhibition of transcription factors to describe gene expression alterations in our data set (Additional file 4: Table S2). In addition, IPA was used to generate networks which are graphical representation of molecular relationships between different genes.

#### Validation of gene expression changes by RT-PCR

**Figure 2:**
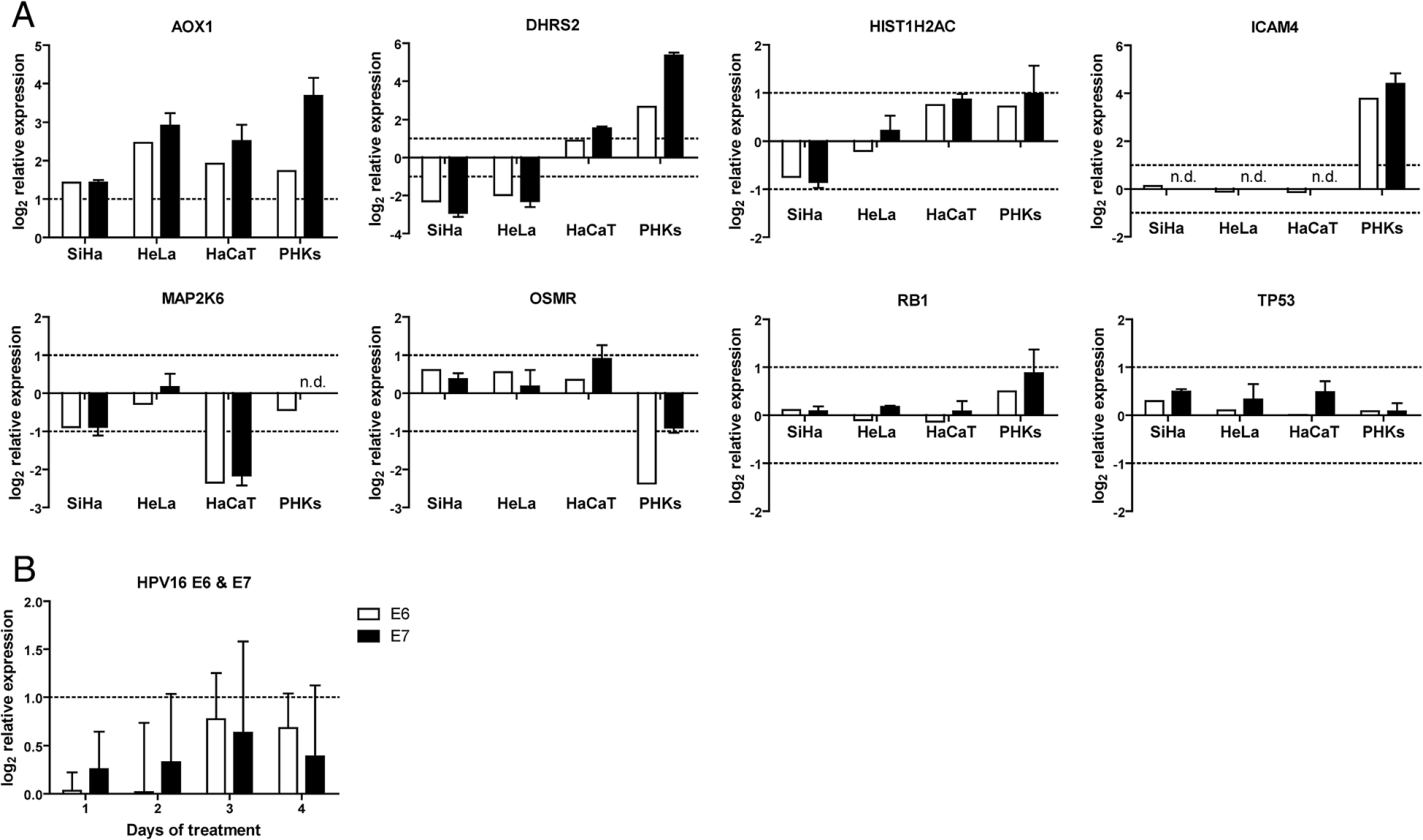
**Gene expression evaluated by real-time RT-PCR.** (**A**) The TaqMan® Fast Universal PCR Master Mix and TaqMan® Gene Expression Assays from Applied Biosystems were employed to determine expression of the following genes (with assay ID’s): AOX1 (Hs00154079_m1), DHRS2 (Hs01061576_m1), HIST1H2AC (Hs00374312_s1), ICAM4 (Hs00169941_m1), MAP2K6 (Hs00992389_m1), OSMR (Hs00384276_m1), TP53 (Hs99999147_m1), and RB1 (Hs01078075_m1) in SiHa, HeLa, HaCaT, and PHKs following CDV treatment for 72 h. White bars represent the relative expression values of the microarray data. Black bars represent average RT-PCR values (±SD), relative to untreated cells and normalized against β–actin, from three independent samples. (**B**) Relative expression levels of HPV16 E6 and E7 in SiHa cells following treatment with CDV for different times, relative to untreated SiHa cells and normalized against β–actin. The bars show average values (±SD) of at least three independent experiments. An absolute 2-fold change difference was considered as biologically significant and is indicated by dashed lines in the figures.

To validate the microarray data, the expression of selected genes (*AOX1*, *DHRS2*, *HIST1H2AC*, *ICAM4*, *MAP2K6*, and *OSMR*) was quantified by real time RT-PCR. Genes that were found to be up- or downregulated by CDV in the microarray data were confirmed by RT-PCR assay (Figure 2A) while those that were not DE in the microarray data showed similar results by RT-PCR. Only a minor difference was observed in the relative expression level of *DHRS2* in HaCaT cells. This gene was 1.9-fold upregulated in the microarray data, which was just below the cut-off (2-fold DE), while being 2.9-fold upregulated in the RT-PCR assay.

Considering that HPV abrogates the functions of the p53 and pRb tumor suppressor proteins and that CDV treatment results in increased levels of these two proteins [19], we also evaluated *TP53* and *RB1* mRNA levels by RT-PCR. Similar to the microarray data, no changes in expression levels of *TP53* and *RB1* were registered by RT-PCR (Figure 2A). Thus, increased p53 and pRb proteins levels following treatment with CDV reflect post-transcriptional regulation of these genes.

### CDV activates the inflammatory response by different mechanisms in immortalized cells and PHKs

**Figure 3:**
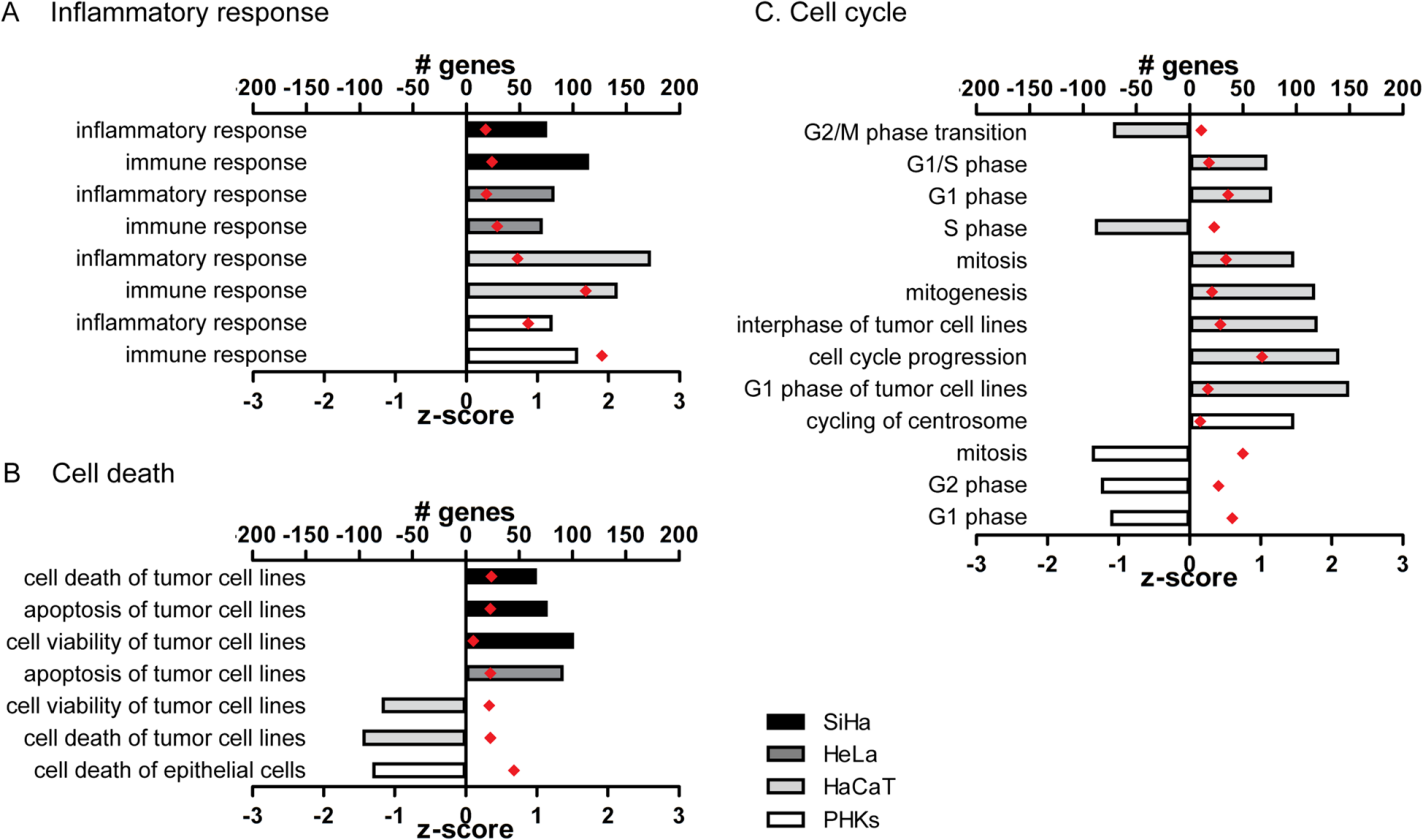
**Effect of CDV exposure on functions related to inflammatory response (A), cell death (B), and cell cycle (C).** The regulation z-score predicts whether an identified biological function is activated or inhibited. Positive z-scores indicate activation of a biological function, while negative z-scores suggest an inhibition. Absolute z-score values above 1 are considered significant. All identified functional annotations are represented in Additional file 2: Figure S2.

A comparison of the functional annotations affected by CDV in either of the four cell types revealed ‘immune response’ and ‘inflammatory response’ to be the only functions upregulated in the different cell types (Figure 3). However, canonical pathway analysis showed that the effect of CDV on immune response pathways is different for immortalized keratinocytes and HPV^+^ tumor cells compared to normal keratinocytes (Additional file 3: Table S1). Despite the lower number of DE genes in immortalized keratinocytes and HPV^+^ tumor cells than in PHKs, a higher proportion of pathways related to immune response was seen in these cells: 3/9 in SiHa, 21/53 in HeLa, 31/57 in HaCaT, compared to 5/35 in PHKs.

Networks were then constructed with DE genes related to inflammatory response (Additional file 5: Figure S3), showing a distinct drug effect on this function in the different cell types. Pathways included in the ‘inflammatory response’ networks showed that CDV modulated several inflammation-associated signaling pathways in immortalized cells and HPV^+^ tumor cells: ‘Acute Phase Response Signaling’ in SiHa, HeLa and HaCaT cells; ‘Activation of IRF by Cytosolic Pattern Recognition Receptors’ , ‘IL-10 Signaling’ , ‘IL-6 Signaling’ , ‘p38 MAPK Signaling’ , ‘TREM1 Signaling’ , ‘Interferon Signaling’ in HeLa and HaCaT cells; ‘ILK Signaling’ , ‘Oncostatin M Signaling’ , and ‘Role of RIG1-like Receptors in Antiviral Innate Immunity’ in HeLa cells; ‘Toll-like Receptor Signaling’ in SiHa cells; and ‘HMGB1 Signaling’ , ‘IL-15 Production’ , ‘IL-17 Signaling’ , ‘IL-8 Signaling’ , ‘NF-κB Signaling’ , and ‘OX40 Signaling’ in HaCaT cells (Additional file 5: Figure S3). In contrast, only two pathways (‘Interferon Signaling’ and ‘Role of IL-17A in Psoriasis’) related to inflammatory response were recognized in PHKs.

Among the DE genes involved in inflammatory response, solely one gene (*AOX1*) was found to be upregulated in all four cell types while *MGLL* was the only gene upregulated in the immortalized keratinocytes and HPV^+^ tumor cells (Additional file 5). Few genes [such as the adhesion molecule *NEDD9* (SiHa and HeLa) and several genes involved in interferon signaling (HaCaT)] were upregulated both in normal keratinocytes and in one of the immortalized cells.

Increased expression of pro-inflammatory cytokines (*IL1*, *IL6*, *IL18*), genes involved in cytokine-cytokine signaling cascades (*IL1R*, *IL6R*, *TNFAIP3*, *TNFAIP6*, *TNFSF13B*, *PTX3*), cell-cell adhesion (*CDH1*, *ICAM1*, *FN1, ITGA2*), tissue remodeling (*VEGF*), extracellular matrix (*ECM*), and proteolysis (*SERPINE1*) characterized the inflammatory response induced by CDV in immortalized keratinocytes and HPV^+^ tumor cells. Also, regulators of cytokine signaling and NF-κB activation (*SPHK1*, *IRAK2*), enzymes involved in the synthesis of prostaglandins (*PTGS1*), deubiquinating enzymes (*CYLD*), and members of the G-protein coupled receptor superfamily (*ADRB2*) were upregulated in these cells. In PHKs, the inflammatory response was mainly driven by upregulation of genes involved in interferon signaling, including *IFIT1*, *IRF1*, *OAS1*, and *STAT1*.

Most of the DE genes in the PHKs ‘inflammatory response’ network were not affected in the other cell types. Moreover, some of the genes in these networks were oppositely affected in PHKs *versus* immortalized keratinocytes and HPV^+^ tumor cells: extracellular matrix protein tenastatin (*TNC*) downregulated in PHKs and upregulated in SiHa and HaCaT cells; topoisomerase *TOP2*, lipoxygenase *ALOX5*, mitogen-activated protein kinase *MAP3K8,* aminopeptidase *ERAP1*, and PDZ binding kinase *PBK* upregulated in PHKs and downregulated in HaCaT cells; transforming growth factor *TGFB2* and transcriptional regulator *NUPR1* upregulated in HaCaT and downregulated in PHKs; myosin light chain kinase *MYLK* upregulated in HeLa cells and downregulated in PHKs.

### Retinoid X receptor (RXR) pathways are distinctly affected by CDV in immortalized cells and PHKs

Retinoid X receptors (RXRs) are nuclear receptors which are ligand-regulated transcription factors that modulate development, differentiation, and homeostasis. They recognize target genes by binding to specific DNA recognition sequences, known as hormone response elements. RXRs are important heterodimer partners for many nuclear receptors, including vitamin D3 receptors (VDRs) and liver X receptors (LXRs) [31].

Activation of LXR/RXR pathways following CDV treatment was exclusively observed in the immortalized keratinocytes and HPV^+^ tumor cells (Additional file 3: Table S1) and was associated with increased mRNA levels of the toll-like receptor *TLR4*, ABC transporters (*ABCG1, ABCA1*), inflammatory cytokines (*IL1A*, *IL6*, *IL18*), cytokine receptors (*IL1R1*, *IL1RN*, *IL1R2*, *IL36RN*), matrix metallopeptidase (*MMP9*), and/or cyclooxygenase (*PTGS2*). Activation of LXR/RXR pathways was also linked to downregulation of genes involved in fatty acid biosynthesis such as *SCD* and of the E3 ubiquitin-protein ligase (*MYLIP*) in HaCaT cells. Except for *ARG2* that was upregulated in HaCaT and downregulated in PHKs, these genes were not affected in PHKs.

In contrast to HPV^+^ cells, activation of the VDR/RXR signaling pathway was recorded in HaCaT and PHKs, yet DE genes implicated in this pathway were rather different between these two cell types. Only increased expression of cystatin *CST6* (implicated in tumor suppression) and of the dehydrogenase *HSD17B2* (involved in sterol metabolism) were common to both PHKs and HaCaT.

### Rho GTPase pathways were affected by CDV exclusively in immortalized keratinocytes and HPV^+^ tumor cells

Pathway analysis showed that changes in Rho GTPase pathways were solely observed in the immortalized cells and HPV^+^ tumor cells: ‘RhoGDI Signaling’ in both HPV^+^ cells; ‘Rac Signaling’ in SiHa cells; ‘RhoA Signaling’ , ‘Regulation of Actin-based Motility by Rho’, and ‘Signaling by Rho Family GTPases’ in HeLa cells; and ‘Cdc42 Signaling’ in HaCaT (Additional file 3: Table S1). Genes upregulated by CDV that were associated with these pathways encompassed: several members of the major histocompatibility (HLA) complex, different receptors (estrogen receptor *ESR1*, NK cell-activating receptor *NCR2*, extracellular matrix receptor *CD44*), several regulators of the Rho family of GTPases [E-cadherin (*CDH1*), integrin alpha-2 (*ITGA2*), myosin (*MYL9, MYLK*), the ras GTPase-activating-like protein *IQGAP2*, the insulin-like growth factor 1 *IGF1*, actins (*ACTA2*, *ACGTG2*, *ACTC1*), the Rho GTPase binding protein *CDC42EP3*] and a member of the Abelson family of nonreceptor tyrosine protein kinases *ABCL2*. Only three genes (the Rho GDP dissociation inhibitor *ARHGDIA*, the transcription factor *FOS*, and the protein kinase *PRKCA*) involved in Rho GTPase pathways were downregulated by CDV in immortalized cells. Except for *MYL9* and *MYLK* that were oppositely regulated in PHKs *versus* immortalized keratinocytes and HPV^+^ tumor cells, none of these genes was DE in normal keratinocytes after CDV exposure. Interestingly, another Rho GDP dissociation inhibitor *ARHGDIB* was upregulated in PHKs.

### Specific gene expression signatures in HPV^+^ tumor cells and immortalized keratinocytes treated with CDV

Four genes (*GLIPR1*, *MAFB*, *MGLL*, and *SERPINB7*) were exclusively induced by CDV in all three immortalized cells (Figure 1B). These genes are involved in cell death (*GLIPR1* and *MAFB*), growth of cells (*SERPINB7*), differentiation (*MAFB*), and migration (*MAFB* and *MGLL*). Furthermore, *MGLL* was associated with lipid metabolism which plays a critical role in malignancy of cancer cells [32] and indeed, lipid metabolism was affected by CDV in HeLa and HaCaT cells (Additional file 2: Figure S2).

Functions related to cancer encompassed the largest number of genes (and most significant *P*-values) in all tested cell types. While a significant z-score for functions related to cancer was calculated in the immortalized cells, functional annotations associated with malignant transformation had a non-significant z-score in PHKs (Additional file 2: Figure S2).

Based on DE of target genes following exposure to CDV, activation or inhibition of transcription factors was predicted by means of upstream regulator analysis with IPA (Additional file 4: Table S2). In SiHa cells, solely MYCN activities showed a significant negative z-score and thus predictive of a decreased activity. Based on z-score values, decreased activities of MYCN were determined in all three immortalized cells although a non-significant *P*-value was calculated for SiHa and HaCaT cells. Activities of the MYC transcription factor, another member of the MYC family of transcription factors, were predicted to be inhibited in HeLa and HaCaT cells.

### Selectivity of CDV for HPV^+^ tumor cells: induction of apoptosis

**Figure 4:**
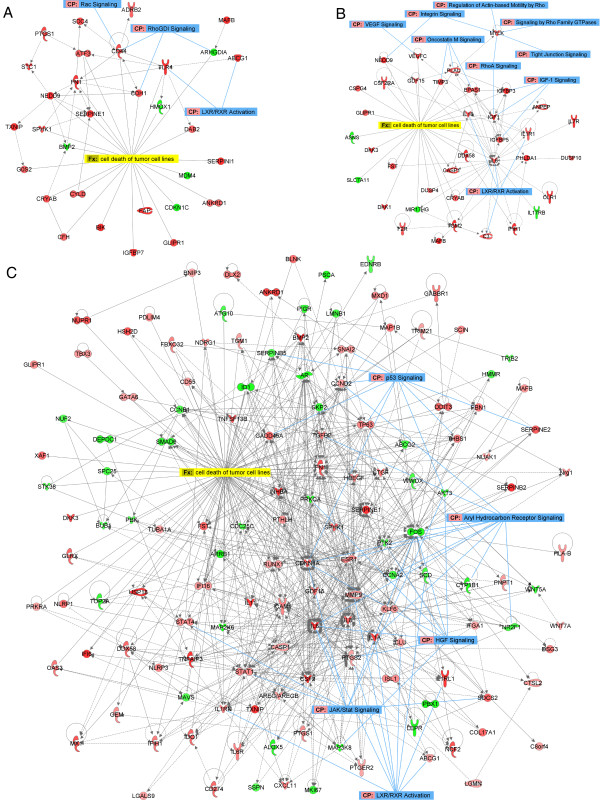
**Networks of ‘cell death of tumor cell lines’ function and corresponding transcripts.** Networks were constructed with IPA software using genes DE and involved in ‘cell death of tumor cell lines’ following CDV treatment of (**A**) SiHa, (**B**) HeLa, or (**C**) HaCaT cells. A network is a graphical representation of the molecular relationships between molecules (nodes). The biological relationship between two nodes is represented as an edge connecting two nodes. All edges are supported by information from the literature stored in the Ingenuity Pathways Knowledge Base. The intensity of the node color indicates the degree of up-regulation (red) or down-regulation (green) following CDV treatment. Canonical pathways identified by IPA in the networks are shown in blue. Except for *DAB2*, *SERPINI1, CFH*, and *NEDD9* in SiHa; *NEDD9*, *DKK1*, *DDX58*, *CDF15*, *DUSP4*, *CASP1*, *IGFBP3*, *F2R*, *SLC7A11*, and *ASNS* in HeLa; *XAF1*, *TRIM21*, *USP18*, *TNFSF13B*, *STAT4*, *GLRX*, *DDX58*, *GEM*, *TBX3*, *BMP2*, *TUBA1A*, *STAT1*, *CTGF*, *IL8*, *GDF15*, *CYP1B1*, *PTHLH*, *THBS1*, *CASP1*, *IL11*, *CLU*, and *CTSL2* in HaCaT; no changes in expression for genes included in the cell death networks built for the immortalized cells were observed in PHKs. Notably, *BMP2* (SiHa); *MYLK* (HeLa); and *NUPR1*, *NUF2*, *SPC25*, *BUB1*, *TOP2A*, *DEPC1*, *CCNB1*, *PBK*, *TGFB2*, *TP63*, *ABCG2*, *CCNA2*, *MKI67*, *SSPN*, *NRG1*, *MAP3K8*, and *ALOX5* (HaCaT) were inversely regulated in PHKs.

The functional annotation ‘apoptosis of tumor cell lines’ was activated following CDV treatment in HPV^+^ cells (Figure 3). Specific sets of genes linked to cell death of tumor cells appeared to be altered following CDV treatment (Figure 4A-B). Most of these genes were only affected in SiHa and/or HeLa cells but not (or reversely) affected in PHKs. Among others, downregulation of *MDM4* and *ARHGDIA* and upregulation of *BIK* and *CYLD* in SiHa cells, and upregulation of *DKK3*, *MYLK*, *PLAU*, and *TIMP3* in HeLa cells, were associated with induction of cell death. Upregulation of *CRYAB* in HPV^+^ cells was linked to both decreased apoptosis and decreased growth of cells, reflecting the diverse effects described for this gene. The association of DE genes with pathways related to apoptosis signaling was highlighted in the cell death networks built for the malignant cells (Figure 4A-B).

In contrast to HPV^+^ cells, HaCaT showed decreased ‘cell death of tumor cells’ and ‘cell viability of tumor cells lines’ following CDV treatment (Figure 3). Pathways affected by CDV identified in the cell death network built for HaCaT were different from those found in HPV^+^ cells and included ‘p53 Signaling’ , ‘Aryl Hydrocarbon Receptor Signaling’ , ‘HGF Signaling’ , and ‘JAK/STAT Signaling’ (Figure 4C).

### CDV affects cell cycle regulation differently in immortalized keratinocytes *versus* normal keratinocytes

Functional analysis suggested distinct effects of CDV on cell cycle in PHKs and HaCaT, while no functional annotations associated with cell cycle were identified in HPV^+^ cells (Figure 3). Similarly, pathways related to cell cycle control were mainly identified in HaCaT and PHKs (Additional file 3: Table S1). Although the activities of the transcription factor p53 were activated in HeLa and HaCaT (Additional file 4: Table S2), the ‘p53 Signaling’ pathway was affected in HaCaT and normal keratinocytes but not in HPV^+^ cells, with *TP63* (member of the p53 transcription factor family) downregulated in PHKs and upregulated in HaCaT.

Distinct sets of genes involved in pathways related to ‘cell cycle’ and ‘DNA replication, recombination, and repair’ were altered in HaCaT and PHKs. Several cyclins and cyclin-dependent kinases (CDKs) that play a key role in cell cycle control were differentially modulated by CDV in HaCaT and PHKs: *CCNA2* and *CCNB1* were downregulated in HaCaT and upregulated in PHKs; *CDK1*, *CDK6*, and *CCNE2* were upregulated in PHKs, but not in HaCaT.

Prediction of transcription factor activities also showed significant differences between PHKs and HaCaT (Additional file 4: Table S2). Notably, SMARCB1 (that relieves repressive chromatin structures) predicted functions were activated in HaCaT, but inhibited in PHKs. TBX2 [that suppresses the expression of CDK inhibitors such as p15 and p21 and destabilizes p53 by suppressing expression of ARF (an inhibitor of MDM2)] predicted functions were inhibited in HaCaT but activated in PHKs. Other transcription factors appeared to be either activated or inhibited exclusively in HaCaT or PHKs, but not in both. Thus, the activities of the tumor suppressor SMARC4A (that mediates ATP-dependent chromatin remodeling processes) and of the histone demethylase KDM5B (that associates with and contributes to the repression of E2f-target genes during senescence) were exclusively activated in HaCaT cells. Furthermore, by inhibiting CDKs, the tumor suppressor p16 (CDKN2A), whose predicted activities were upregulated in HaCaT cells, triggers the G1-S checkpoint that is often considered to be crucial for inducing a senescence-like growth arrest. In line with growth arrest in HaCaT cells, are the decreased predicted activities of the E2f transcription factor (regulator of expression of genes such as cyclins, CDKs, components of the pre-replication, and DNA synthesis genes) and the increased predicted activities of the chromatin associated protein HMGB1 (that facilitates p53 phosphorylation after to genotoxic stress) and of NFκB (a pleiotropic transcription factor that can be activated by DNA damage).

The occurrence of cell cycle arrest in HaCaT was further evidenced by upregulation of *CDKN1A* (p21) and downregulation of *CCNA2*, *CCNB1*, *TOPA2*, *SKP2*, *HDAC8*, and *PPM1L*, in contrast to PHKs (Additional file 6: Table S3).

### Specific gene expression signatures in PHKs exposed to CDV

#### Activation of metabolic pathways

Whereas immortalized keratinocytes and HPV^+^ tumor cells were found to have more alterations in immune response pathways compared to the PHKs, seventeen different pathways (out of 35) linked to metabolism were seen in PHKs *versus* only one (SiHa), two (HaCaT) and three (HeLa) in CDV-treated immortalized cells (Additional file 3: Table S1).

#### DNA damage response and survival of epithelial cells

Pathways related to DNA repair (‘ATM Signaling’ and ‘DNA Double-Strand Break Repair by Homologous Recombination’) were exclusively identified in PHKs, suggesting activation of DNA repair mechanisms following CDV-induced DNA damage. Several cell division cycle (CDC) homologs (such as *CDC2*, *CDC6*, *CDC7*, *CDC23*, and *CDC25A*), that play important roles in cell cycle transition and DNA replication (Additional file 6: Table S3), were exclusively upregulated in PHKs. In contrast, *CDC25C* was found downregulated in HaCaT. Expression of genes encoding for proteins involved in DNA repair and checkpoint control (including *ANAPC7*, *BRCA1*, *CDT1*, *CKS2*, *EME1*, *GEN1*, *KAT2B*, *KIF11*, *MDM2*, *NBN*, *ORC6*, *PCNA*, *RAD51*, and *RFC3*) were solely upregulated in PHKs (Additional file 6: Table S3).

Importantly, functional analysis revealed a decrease (z-score < 1) of ‘cell death of epithelial cells’ following CDV treatment of PHKs, in contrast to increased (z-score > 1) ‘cell death of tumor cell lines’ in SiHa and HeLa (Figure 3). The upregulation of anti-apoptotic genes (such as *BIRC5*) in PHKs suggested a successful response to DNA damage.

## Discussion

In this study, the basis for selectivity of CDV for HPV^+^ tumor cells could be demonstrated based on analysis of drug incorporation into genomic DNA as well as gene expression profiling in HPV^+^ tumor cells, HPV^-^ immortalized keratinocytes and normal keratinocytes. Bioinformatics analysis of microarray data highlighted distinct responses to CDV exposure in PHKs compared to HPV^+^ cervical carcinoma cells, on one hand, and to HPV^-^ immortalized keratinocytes, on the other hand.

Our findings indicate that the selectivity of CDV for HPV-transformed cells is based on differences in response to DNA damage, replication rate and CDV incorporation into cellular DNA between immortalized cells and PHKs, rather than a specific effect of the drug on the viral oncogenes. However, the presence of E6 and E7 indirectly contributes to the efficacy and selectivity of CDV, because viral oncoproteins deregulate cell cycle, impeding cell cycle checkpoints and DNA repair, thus favoring the antiproliferative effects of CDV.

**Figure 5:**
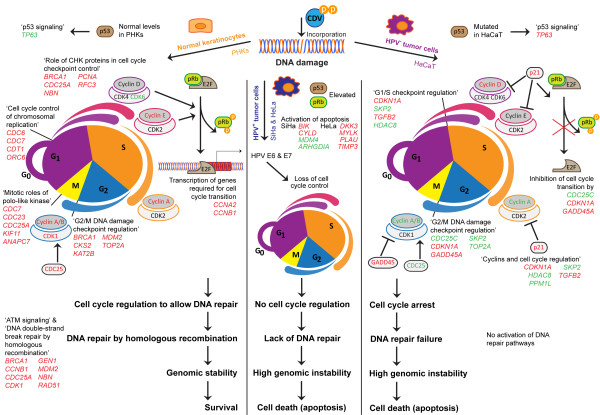
**Differential responses to CDV treatment of HPV^+^ tumor cells and immortalized keratinocytes *versus *PHKs and proposed CDV selectivity mechanism.** Incorporation of CDV during DNA synthesis leads to DNA damage signaling and elevated p53 levels. Distinct responses are elicited depending on the cell type and p53 status. Normal keratinocytes activate cell cycle regulation mechanisms that allow DNA repair by means of homologous recombination, leading to genomic stability and cell survival. Despite blocking cell cycle progression, HPV^-^ immortalized cells are not able to activate a DNA repair mechanism, while HPV^+^ tumor cells lack cell cycle regulation and DNA repair due to E6 and E7 oncoproteins. The inability to repair DNA damage leads to genomic instability and activation of apoptosis in these cells. Altered cell cycle regulation and/or DNA damage repair pathways are cited, with their relevant genes marked in red when upregulated and in green when downregulated.

Gene expression profiling of CDV-treated HaCaT and PHKs revealed specific signatures that clearly explain a differential outcome in both cell types following drug exposure (Figure 5). Except for *CYP1B1* and *THBS1*, complete different sets of genes in pathways related to ‘cell cycle’ and ‘DNA replication, recombination, and repair’ were modulated following CDV exposure of HaCaT and PHKs, supporting a differential effect on ‘cell cycle’ functions in immortalized and normal keratinocytes. Interestingly, mRNA levels of many genes involved in these functions were oppositely regulated by CDV in PHKs and in HaCaT cells or exclusively affected in one of the cell types. HaCaT cells respond to CDV by attempting cell cycle regulation which fails because of the inability of these cells to repair DNA damage (Figure 5). This is further sustained by CDV triggering of ‘p53 Signaling’ in HaCaT and normal keratinocytes but not in cervical cancer cells. Also, the prediction of transcription factor activities points to cell cycle arrest in HaCaT but not in PHKs.

Specific signatures identified in CDV-treated PHKs point to cell cycle regulation and activation of DNA double-strand breaks (DSBs) repair mechanism (‘ATM Signaling’ and ‘DNA Double-Strand Break Repair by Homologous Recombination’), suggesting that CDV can generate DSBs. Homologous recombination (HR) is a conservative process that tends to restore the original DNA sequence at the site of damage. Expression of genes involved in DNA repair by non-homologous end-joining (NHEJ) was not seen in CDV-treated PHKs. This points to a non-mutagenic CDV effect as NHEJ can be mutagenic because it mediates repair by directly ligating the ends of DSBs together, in contrast to HR that is considered a faithful DNA repair process [33]. Since CDV induces accumulation of tumor cells in the S-phase [19,20], and CDVpp, an analogue of deoxycytidine-triphosphate, can be incorporated into cellular DNA, this drug can cause potentially lethal chromosomal DSBs during DNA replication.

In contrast to normal cells that possess an arsenal of repair pathways and cell cycle checkpoints to detect and repair DNA damage, cancer cells as well as immortalized keratinocytes have a significantly reduced set of DNA repair pathways for survival, which can be targeted to develop improved treatment strategies [34]. Differences in the response of normal cells and cancer cells to DNA damaging agents also explain the mechanisms by which the nucleoside analogue ganciclovir induces cell death in tumor cells genetically modified to express the herpes simplex virus thymidine kinase gene [35,36].

Here, we identified DE genes linked to cell death and confirmed at the gene expression level apoptosis induction by CDV [19,21]. It should be noted that apoptosis induction, accumulation of the cells in the S-phase, increased protein levels of the tumor suppressor proteins p53 and pRb, and decreased cell viability were evidenced following exposure of tumor cells to CDV for 4 to 5 days [19,20], indicating that cells need to accumulate sufficient drug-induced stress before apoptosis takes place. Distinct sets of genes linked to cell death were altered following 72 h CDV treatment of SiHa and HeLa cells, suggesting that although CDV treatment leads to apoptosis in malignant cells, different cells may respond to CDV by modulating distinct sets of genes, most likely reflecting variations in the genetic background between tumor cells. Considering the DE genes involved in cell cycle control and cell death in HaCaT, it can be assumed that apoptosis will be triggered at a later time point than in HPV^+^ cells.

HPV^+^ cells, that are more susceptible to the antiproliferative effects of CDV than HPV^-^ immortalized keratinocytes and normal keratinocytes [18], divide very rapidly, present a high genomic instability and are defective in cell cycle control and DNA repair mechanisms due to the expression of E6 and E7 oncoproteins. Thus, CDV treatment of cervical cancer cells may result in significant DNA damage during the S-phase that should be responsible for induction of p53 and apoptosis [19,20].

Some reports claimed that CDV could specifically affect mRNA levels of E6 and E7 [21,37]. Abdulkarim and colleagues found decreased E6 and E7 mRNA levels and reduced protein expression in HPV18 positive cells [21]. However, we were unable to detect E6 protein levels in cervical carcinoma cells, largely due to low endogenous levels of E6, as well as poor quality of available anti-E6 antibodies, in agreement with several reports [38-41]. On the other hand, we did not find a significant alteration in E6 and E7 mRNA levels by quantitative RT-PCR following treatment with CDV at 50 μg/ml for 1- to 7 days (Figure 2B). The elevated p53 and pRb protein levels (reported by [19,21]) cannot be attributed to increased mRNA expression of these genes according to our microarray and RT-PCR data. It appears that the higher p53 protein levels are the consequence of the DNA damage response following CDV treatment that affects the expression of regulators of p53 (such as *MDM2*, *MDM4*, and *CDKN1A*) resulting in a rapid stabilization of p53 via blocking of its degradation. This is in agreement with previous reports of post-transcriptional regulation of these genes [42], showing a rapid increase in p53 protein concentration without de novo transcription which is particularly advantageous in cells with severely damaged genomes [43].

*MDM2* and *MDM4* are considered the main cellular antagonist of p53 by limiting its functions [44]. *MDM4*, that inhibits p53 by binding its transcriptional activation domain [45], was downregulated in CDV-treated SiHa cells while *MDM2* was upregulated in CDV-exposed PHKs. Thus, in PHKs, *MDM2* is expected to ubiquitinate p53 and mediate its degradation by nuclear and cytoplasmatic proteasomes. In contrast, in CDV-exposed malignant cells, as a consequence of DNA damage accumulation, stabilization of p53 and induction of several pro-apoptotic genes (such as *BIK*, *CYLD*, *DKK3*, *PLAU*, and *TIMP3*) take place. Activation of *BIK* through transcriptional pathways (dependent on factors such as E2f and p53 or by epigenetic regulating mechanisms) was described following treatment with anti-cancer drugs [46], and upregulation of *BIK* is considered as an interventional approach to treat some tumors [47]. The tumor suppressor *CYLD* encodes for a deubiquitinase that plays a critical role in the regulation of NF-κB and activation of caspase-8, its activation being regarded as a therapeutic target in the treatment of cancers [48]. The tumor suppressor *DKK3* (RIG) induces apoptosis through mitochondrial pathways in human colon cancer [49] and pro-apoptotic actions of *PLAU* in tumor cells have also been described [50]. The tissue inhibitor of metalloproteinases *TIMP3* promotes apoptosis involving stabilization of cell death receptors and activation of caspase-8 [51].

Pro-apoptotic activities have been described for *GLIPR1* and *MAFB* that were upregulated in immortalized keratinocytes and HPV^+^ tumor cells. GLIPR1 was shown to induce apoptosis in prostate cancer [52], and to promote MYC ubiquitination and degradation leading to suppression of cancer development [53]. In line with this report, not only upregulation of *GLIPR1* but also downregulation of the predicted activities of MYC family members were observed in immortalized cells. Maf proteins were shown to possess tumor suppressor activities through induction of expression of the cell-cycle inhibitor p27 [54] and pro-apoptotic activities through inhibition of MYB [55,56] or induction of p53 transcription [57]. MYCN together with MYB were shown to be involved in a reciprocal regulatory loop promoting survival/proliferation of neuroblastoma cells [58,59]. Both transcription factors are considered potential specific targets for cancer therapy and downregulation of MYCN expression by treatment with antisense or by retinoid acids decreases proliferation of neuroblastoma cells [60]. Several miRNAs, including miR-17-92, are also known to be regulated by MYCN [61], which showed reduced predicted activities in HeLa. MYCN expression was found to be inversely correlated with *DKK3* expression [62], which is in line with our HeLa data. Although CDV did not affect MYCN expression, decreased predicted activities of this proto-oncogene support the antiproliferative effects of CDV and apoptosis induction. Activities of MYC members were also reported to be altered by a few conventional cytotoxic drugs that target microtubules, topoisomerases, or DNA, RNA and protein synthesis [63].

Several cyclins and CDKs were differentially modulated by CDV in HPV^-^ cells (Figure 5). Increased transcription of genes required for cell cycle progression (*CCNA2* and *CCNB1*) suggests that pRb can be phosphorylated in PHKs leading to release of E2f. Furthermore, cell cycle progression appeared to be blocked in HaCaT cells as evidenced by upregulation of *CDKN1A* (p21) that blocks the activity of cyclin-CDK2/4 complexes and GADD45A, whose transcript levels are increased following stressful growth arrest by treatment with DNA-damaging agents. As a consequence of the increased expression of *CDKN1A*, the complexes cyclinD-CDK4/6 and cyclinE-CDK2 are not activated and pRb cannot be phosphorylated in order to release E2f.

Only two genes (*AOX1* and *CLIC3*) were common to all four cell types. Altered expression of *CLIC3* following CDV exposure was not associated with any of the functions or pathways modulated by CDV. In contrast, *AOX1* was linked to inflammatory response, the only common function found activated in all cell types. However, distinct pathways linked to inflammatory response were affected by CDV in immortalized keratinocytes and HPV^+^ tumor cells *versus* PHKs. Importantly, ‘Acute Phase Response Signaling’, a rapid inflammatory response using non-specific defense mechanisms that provides protection not only against microorganisms but also to tissue injury, neoplastic growth or immunological disorders [64], was exclusively identified in SiHa, HeLa and HaCaT cells. Induction of DNA damage by CDV in immortalized cells was associated with acute phase response signaling which is in agreement with data showing that DNA damage leads to an upregulation of immunostimulatory surface ligands and to an increased secretion of pro-inflammatory cytokines in senescent cells [65]. This may result in the activation of acute response signaling in CDV-exposed immortalized cells that may be important *in vivo* for clearance of the senescent cells. Considering the number of pathways linked to immune response identified in the CDV-treated immortalized cells, it can be inferred that the inflammatory response plays a crucial role in the response of tumor cells to CDV and that activation of the inflammatory response can be regarded as a cellular reaction to CDV-induced stress.

LXRs play a key role in cholesterol transport by inducing the expression of ATP-binding cassette (ABC) transporters involved in cholesterol efflux. These nuclear receptors also control diverse pathways implicated in development, reproduction, metabolism, immunity and inflammation. Recent insights into LXR signaling revealed that targeting activation of the LXR pathway harbor promises for the management of metabolic disorders, chronic inflammatory diseases, cancer, and neurodegenerative diseases [66,67]. Therefore, activation of LXR/RXR by CDV in immortalized cells might be an important mediator in the inflammatory response induced by CDV in these cells.

Also, Rho GTPase pathways were exclusively identified in immortalized keratinocytes and HPV^+^ tumor cells. Rho GTPase proteins (RhoA-C, Rac1, and Cdc42) function as molecular switches in a variety of signaling pathways following stimulation of cell surface receptors and regulate several biological processes, including cell cycle control, epithelial cell polarity, cell migration, cell survival and angiogenesis [68]. Modulation of Rho GTPase pathways by CDV identified in our microarray data is consistent with a previous report that demonstrated the efficacy of CDV in disrupting invasion of HeLa cells by decreasing CXCR4 expression and inhibiting Rho/ROCK activation [23]. RhoGDP dissociation inhibitors (RhoGDIs) are considered antiapoptotic molecules [69], and different therapeutic strategies that target RhoGDIs have previously been proposed [70]. Thus, modulation of the RhoGDI and Rac signaling pathways by CDV may be important in induction of cell death as evidenced by downregulation of *ARHGDIA* (one of the RhoGDIs) in SiHa cells.

## Conclusion

In summary, cell cycle checkpoint control and DNA damage repair occur only in PHKs following CDV treatment. HPV^+^ cells are more susceptible to the antiproliferative action of CDV because they are completely unable to respond to CDV-induced stress while HaCaT cells still can respond via induction of several signaling pathways but they lack proper cell cycle checkpoint and DNA repairing mechanisms. Furthermore, gene expression profiling allowed the identification of several pathways and functions induced or repressed following exposure to CDV that were different in PHKs compared to HPV^+^ and/or HPV^-^ cells, including Rho GTPase pathways and ‘acute phase response’ exclusively activated in immortalized cells. Our data also have implications for the use of CDV in combination with standard therapy for the treatment of cancer cells that rapidly divide and that show a defect in DNA repairing mechanisms. CDV-induced DNA damage will preferentially accumulate in the tumor cells resulting in S-phase arrest and cell death. Moreover, our findings help to explain the selective effect of CDV which has been clearly documented in several case reports and phase II/III clinical studies [11,14-16]. CDV has been used mostly topically to treat HPV-associated diseases showing a selective antiproliferative effect against HPV lesions without being associated with local side-effects on neighboring normal epithelial cells.

The present findings may lay the scientific basis for further studies on functions and pathways found to be differentially affected by CDV in immortalized keratinocytes and HPV^+^ tumor cells *versus* normal keratinocytes. Furthermore, this detailed microarray analysis generated a source of novel molecular targets for the treatment of HPV-associated diseases and potentially of non-HPV neoplasias. Our data revealed not only interactions between genes that illustrate useful pathways for new therapeutic targets but also for understanding the mechanism of selectivity of CDV. Additional combined genomic and proteomic studies are required to reveal in even more detail the precise mode of action of CDV and related acyclic nucleoside phosphonates as double-acting (antiviral and antiproliferative) drugs.

## Competing interests

The authors declare they have no competing interests.

## Author contributions

TDS, GA, DT, LN, and RS conceived and designed the experiments. TDS performed the experiments. TDS, GA, DT, LN, and RS analyzed the data. TDS, GA, DT, and RS were responsible for drafting the article. All authors critically revised the article and finally approved the manuscript prior to publication.

## Pre-publication history

The pre-publication history for this paper can be accessed here:

http://www.biomedcentral.com/1755-8794/6/18/prepub

## Supplementary Material

Additional file 1 **Normalization, filtering, and analysis of microarray data derived from CDV treated and untreated cells.** Flow chart of the microarray experiments that was applied to obtain our data sets. Click here for file

Additional file 2 **Functional annotations upregulated or downregulated within functional categories following CDV treatment in different cell types.** For the functional analysis, categories associated with unrelated cell types and with irrelevant diseases were omitted. The criteria for selection of functional annotations were based on z-score and statistical significance (P-value < 0.05). The regulation z-score predicts whether an identified biological function is activated or inhibited. Positive z-scores indicate activation of a biological function, while negative z-scores suggest an inhibition. Absolute z-score values above 1 are considered significant. Click here for file

Additional file 3 **Canonical pathway analysis.** Canonical pathway analysis of changes in gene expression following incubation of SiHa, HeLa, HaCaT, and PHKs with CDV for 72 h. The significance of the associations between the genes from the data sets and the canonical pathways were determined based on two parameters: (i) P-value, calculated by the Fischer’s exact test, that determines the probability that there is an association between the genes in the data set and the canonical pathway that cannot be explained by chance alone and (ii) by the ratio of the number of genes from the data set in a given pathway divided by the total number of molecules in the given canonical pathway. P-values < 0.05 were considered statistically significant. Pathways marked in black in the PHKs data represent the pathways that were exclusively identified in these cells, while pathways marked in grey represent the pathways that were shared among SiHa, HeLa cells and/or HaCaT cells. Click here for file

Additional file 4 **Prediction of transcription factor activities in the different cell types.** The activity of the transcription factors was evaluated by P-value and regulation z-score whose calculation is based on relationships with their target genes. The relationships represent experimentally observed gene expression or transcription events associated with a direction of change that result in activation of inhibition (as derived from the literature compiled in the IPKB). The z-score predicts the identified transcription factors to be activated (positive z-score) or inhibited (negative z-score). Only upstream regulators that showed an absolute z-score > 2 in at least one of the four cell types are represented. P-values <0.05 were considered significant. Click here for file

Additional file 5 **Inflammatory response networks.** Networks were constructed with IPA software using genes DE and involved in ‘inflammatory response’ following CDV treatment of (A) SiHa, (B) HeLa, (C) HaCaT, or (D) PHKs. Click here for file

Additional file 6 **Effect of CDV on ‘cell cycle’ and ‘DNA replication, recombination, and repair’ in HPV**^**- **^**cells.** Genes modulated by CDV in HaCaT and/or PHKs that are involved in pathways related to ‘cell cycle’ and ‘DNA replication, recombination, and repair’. Click here for file
