# Supplementary material for: Cidofovir selectivity is based on the different response of normal and cancer cells to DNA damage
Source: BMC Med Genomics. 2013 May 23;6:18. doi: 10.1186/1755-8794-6-18 (PMC3681722; doi:10.1186/1755-8794-6-18)

Additional file 1. Normalization, filtering, and analysis of microarray data derived from CDV treated and untreated cells.
Flow chart of the microarray experiments that was applied to obtain our data sets.

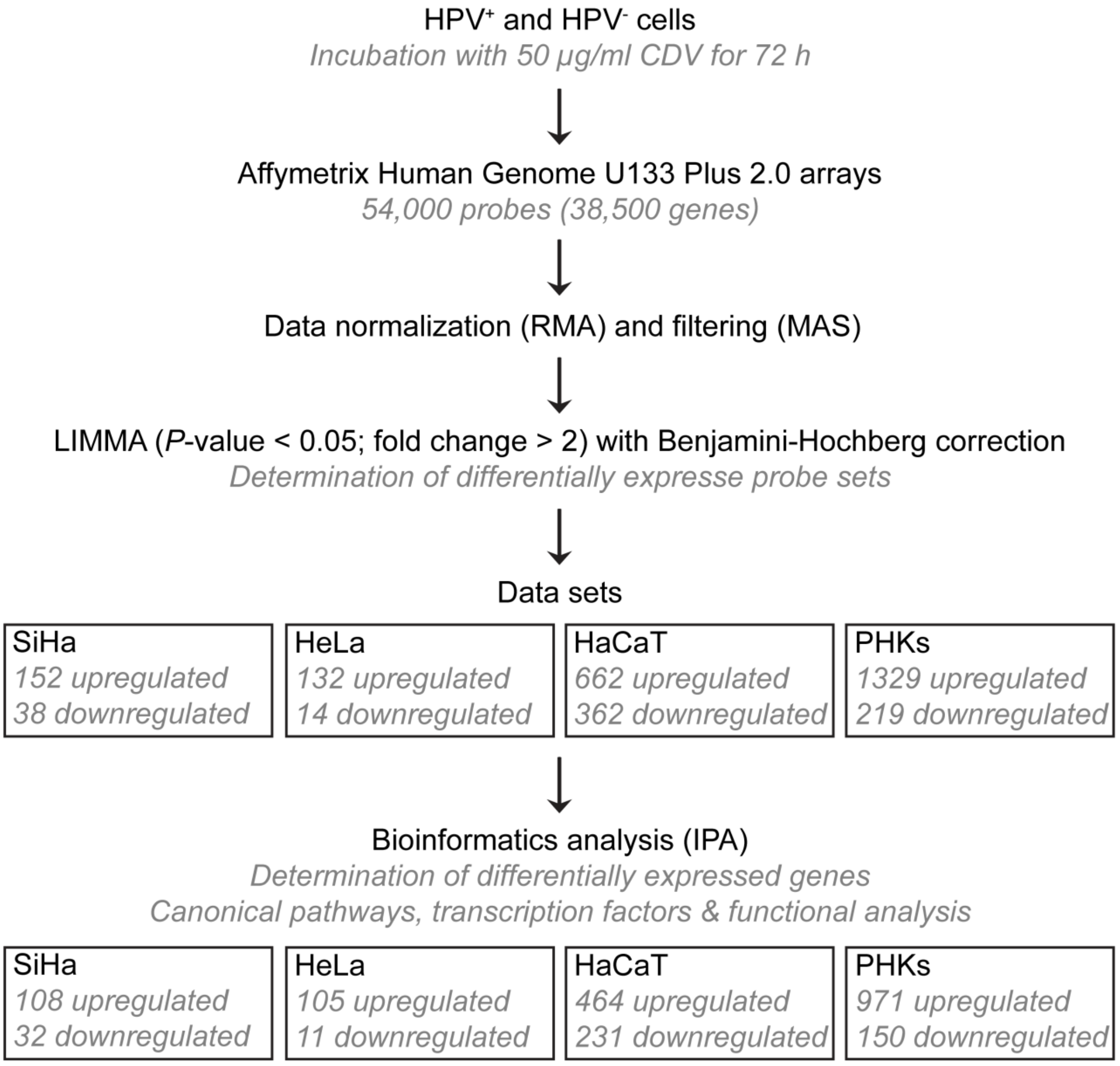

Supplement: Additional file 1 — Normalization, filtering, and analysis of microarray data derived from CDV treated and untreated cells. Flow chart of the microarray experiments that was applied to obtain our data sets. [file 1755-8794-6-18-S1.pdf]
