# Supplementary material for: Cidofovir selectivity is based on the different response of normal and cancer cells to DNA damage
Source: BMC Med Genomics. 2013 May 23;6:18. doi: 10.1186/1755-8794-6-18 (PMC3681722; doi:10.1186/1755-8794-6-18)

Additional file 2. Functional annotations upregulated or downregulated within functional categories following CDV treatment in different cell types.
For the functional analysis, categories associated with unrelated cell types and with irrelevant diseases were omitted. The criteria for selection of functional annotations were based on z-score and statistical significance (P-value < 0.05). The regulation z-score predicts whether an identified biological function is activated or inhibited. Positive z-scores indicate activation of a biological function, while negative z-scores suggest an inhibition. Absolute z-score values above 1 are considered significant.

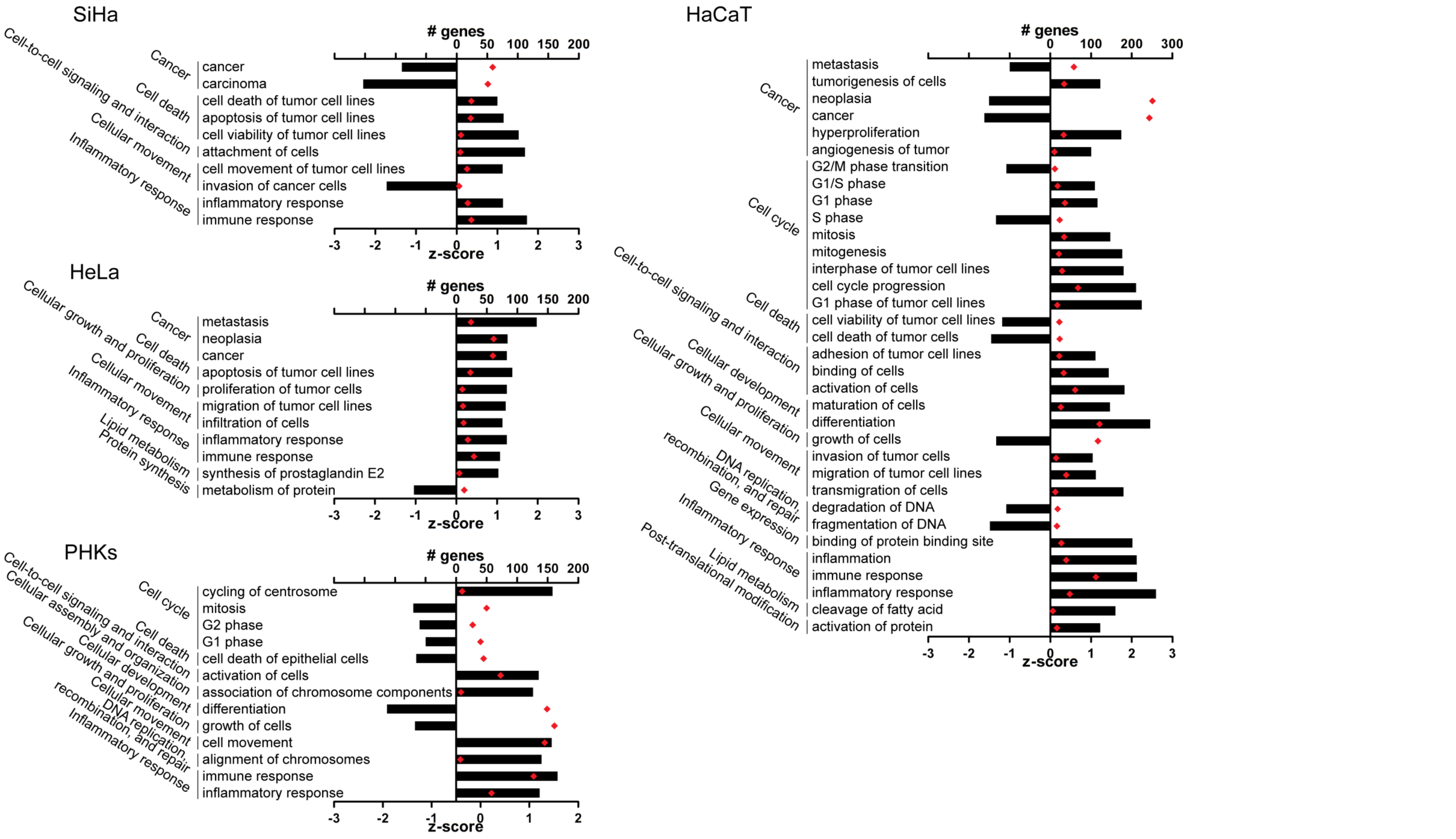

Supplement: Additional file 2 — Functional annotations upregulated or downregulated within functional categories following CDV treatment in different cell types. For the functional analysis, categories associated with unrelated cell types and with irrelevant diseases were omitted. The criteria for selection of functional annotations were based on z-score and statistical significance (P-value < 0.05). The regulation z-score predicts whether an identified biological function is activated or inhibited. Positive z-scores indicate activation of a biological function, while negative z-scores suggest an inhibition. Absolute z-score values above 1 are considered significant. [file 1755-8794-6-18-S2.pdf]
