# Supplementary material for: Cidofovir selectivity is based on the different response of normal and cancer cells to DNA damage
Source: BMC Med Genomics. 2013 May 23;6:18. doi: 10.1186/1755-8794-6-18 (PMC3681722; doi:10.1186/1755-8794-6-18)
Supplement: Additional file 3 — Canonical pathway analysis. Canonical pathway analysis of changes in gene expression following incubation of SiHa, HeLa, HaCaT, and PHKs with CDV for 72 h. The significance of the associations between the genes from the data sets and the canonical pathways were determined based on two parameters: (i) P-value, calculated by the Fischer’s exact test, that determines the probability that there is an association between the genes in the data set and the canonical pathway that cannot be explained by chance alone and (ii) by the ratio of the number of genes from the data set in a given pathway divided by the total number of molecules in the given canonical pathway. P-values < 0.05 were considered statistically significant. Pathways marked in black in the PHKs data represent the pathways that were exclusively identified in these cells, while pathways marked in grey represent the pathways that were shared among SiHa, HeLa cells and/or HaCaT cells. [file 1755-8794-6-18-S3.docx]

**Additional file 3. Canonical pathway analysis.**

Canonical pathway analysis of changes in gene expression following incubation of SiHa, HeLa, HaCaT, and PHKs with CDV for 72h. The significance of the associations between the genes from the data sets and the canonical pathways were determined based on two parameters: (i) *P*-value, calculated by the Fischer’s exact test, that determines the probability that there is an association between the genes in the data set and the canonical pathway that cannot be explained by chance alone and (ii) by the ratio of the number of genes from the data set in a given pathway divided by the total number of molecules in the given canonical pathway. *P*-values < 0.05 were considered statistically significant. Pathways marked in black in the PHK data represent the pathways that were exclusively identified in these cells while pathways marked in grey represent the pathways that were shared among SiHa and HeLa cells or among the three tumor cell lines.

|  | **Ingenuity Canonical Pathways** | **p-value** | **ratio** | **function** |
| --- | --- | --- | --- | --- |
| **PHKs** |  |  |  |  |
|  | Role of IL-17A in Psoriasis | <0.001 | 0.54 | immune response |
|  | p53 Signaling | <0.001 | 0.18 | cell cycle/cell death |
|  | Fatty Acid Metabolism | 0.001 | 0.08 | metabolism |
|  | IL-17A Signaling in Gastric Cells | 0.002 | 0.24 | immune response |
|  | Nicotinate and Nicotinamide Metabolism | 0.003 | 0.09 | metabolism |
|  | Pyrimidine Metabolism | 0.003 | 0.07 | metabolism |
|  | Glutamate Metabolism | 0.003 | 0.09 | metabolism |
|  | Pantothenate and CoA Biosynthesis | 0.004 | 0.08 | metabolism |
|  | Arachidonic Acid Metabolism | 0.005 | 0.06 | metabolism |
|  | Aryl Hydrocarbon Receptor Signaling | 0.005 | 0.09 | cell cycle/cell death |
|  | Role of IL-17F in Allergic Inflammatory Airway Diseases | 0.005 | 0.15 | immune response |
|  | DNA Double-Strand Break Repair by Homologous Recombination | 0.006 | 0.24 | DNA repair |
|  | Hepatic Fibrosis / Hepatic Stellate Cell Activation | 0.008 | 0.10 | disease specific |
|  | Cell Cycle: G2/M DNA Damage Checkpoint Regulation | 0.008 | 0.14 | cell cycle |
|  | Role of CHK Proteins in Cell Cycle Checkpoint Control | 0.008 | 0.17 | cell cycle |
|  | Aminoacyl-tRNA Biosynthesis | 0.008 | 0.08 | metabolism |
|  | VDR/RXR Activation | 0.009 | 0.12 | cellular development |
|  | Bile Acid Biosynthesis | 0.013 | 0.07 | metabolism |
|  | Alanine and Aspartate Metabolism | 0.014 | 0.07 | metabolism |
|  | Cell Cycle Control of Chromosomal Replication | 0.014 | 0.16 | cell cycle |
|  | Tryptophan Metabolism | 0.014 | 0.05 | metabolism |
|  | One Carbon Pool by Folate | 0.017 | 0.11 | metabolism |
|  | Protein Ubiquitination Pathway | 0.018 | 0.08 | protein degradation |
|  | ATM Signaling | 0.022 | 0.13 | DNA repair |
|  | Interferon Signaling | 0.028 | 0.14 | immune response |
|  | Folate Biosynthesis | 0.030 | 0.04 | metabolism |
|  | β-alanine Metabolism | 0.031 | 0.06 | metabolism |
|  | Selenoamino Acid Metabolism | 0.032 | 0.07 | metabolism |
|  | Linoleic Acid Metabolism | 0.035 | 0.07 | metabolism |
|  | Glycine, Serine and Threonine Metabolism | 0.035 | 0.05 | metabolism |
|  | Mitotic Roles of Polo-Like Kinase | 0.037 | 0.11 | cell cyle |
|  | Coagulation System | 0.040 | 0.13 | cardiovascular system |
|  | Valine, Leucine and Isoleucine Degradation | 0.041 | 0.06 | metabolism |
|  | Communication between Innate and Adaptive Immune Cells | 0.042 | 0.07 | immune response |
|  | Factors Promoting Cardiogenesis in Vertebrates | 0.047 | 0.09 | tissue development |
|  |  |  |  |  |
|  |  |  |  |  |
| **SiHa** |  |  |  |  |
|  | Granzyme A Signaling | 0.006 | 0.10 | immune response |
|  | Hepatic Fibrosis / Hepatic Stellate Cell Activation | 0.017 | 0.03 | disease specific |
|  | Acute Phase Response Signaling | 0.017 | 0.02 | immune response |
|  | Sertoli Cell-Sertoli Cell Junction Signaling | 0.034 | 0.02 | cellular growth |
|  | RhoGDI Signaling | 0.035 | 0.02 | Rho GTPase pathways |
|  | Rac Signaling | 0.037 | 0.02 | Rho GTPase pathways |
|  | Fructose and Mannose Metabolism | 0.037 | 0.01 | metabolism |
|  | Toll-like Receptor Signaling | 0.048 | 0.04 | immune response |
|  | LXR/RXR Activation | 0.048 | 0.02 | (lipid) metabolism |
|  |  |  |  |  |
|  |  |  |  |  |
|  |  |  |  |  |
|  | **Ingenuity Canonical Pathways** | **p-value** | **ratio** | **function** |
| **HeLa** |  |  |  |  |
|  | Hepatic Fibrosis / Hepatic Stellate Cell Activation | <0.001 | 0.06 | disease specific |
|  | RhoA Signaling | <0.001 | 0.06 | Rho GTPase pathways |
|  | Role of Pattern Recognition Receptors in Recognition of Bacteria and Viruses | <0.001 | 0.06 | immune response |
|  | ILK Signaling | <0.001 | 0.04 | immune response |
|  | Clathrin-mediated Endocytosis Signaling | <0.001 | 0.04 | cellular immune respons |
|  | Regulation of Actin-based Motility by Rho | <0.001 | 0.05 | Rho GTPase pathways |
|  | Integrin Signaling | <0.001 | 0.03 | cell cycle/cellular growth |
|  | Crosstalk between Dendritic Cells and Natural Killer Cells | <0.001 | 0.05 | immune response |
|  | Tight Junction Signaling | <0.001 | 0.04 | cell cycle/cell death |
|  | Activation of IRF by Cytosolic Pattern Recognition Receptors | <0.001 | 0.06 | immune response |
|  | Caveolar-mediated Endocytosis Signaling | 0.001 | 0.05 | immune response |
|  | Cellular Effects of Sildenafil (Viagra) | 0.001 | 0.03 | disease specific |
|  | Oncostatin M Signaling | 0.001 | 0.09 | immune response |
|  | Actin Cytoskeleton Signaling | 0.001 | 0.03 | organismal growth |
|  | Role of Macrophages, Fibroblasts and Endothelial Cells in Rheumatoid Arthritis | 0.001 | 0.02 | disease specific |
|  | Role of Osteoblasts, Osteoclasts and Chondrocytes in Rheumatoid Arthritis | 0.001 | 0.03 | disease specific |
|  | VEGF Signaling | 0.001 | 0.04 | cellular growth |
|  | Mechanisms of Viral Exit from Host Cells | 0.001 | 0.07 | pathogen-influenced signaling |
|  | Signaling by Rho Family GTPases | 0.002 | 0.02 | Rho GTPase pathways |
|  | MSP-RON Signaling Pathway | 0.002 | 0.06 | immune response |
|  | IGF-1 Signaling | 0.002 | 0.04 | cellular growth |
|  | Paxillin Signaling | 0.002 | 0.04 | organismal growth |
|  | TREM1 Signaling | 0.003 | 0.05 | immune response |
|  | LXR/RXR Activation | 0.004 | 0.03 | (lipid) metabolism |
|  | Glioma Invasiveness Signaling | 0.004 | 0.05 | cancer |
|  | Agrin Interactions at Neuromuscular Junction | 0.006 | 0.04 | nervous system |
|  | IL-10 Signaling | 0.007 | 0.04 | immune response |
|  | Germ Cell-Sertoli Cell Junction Signaling | 0.011 | 0.02 | cellular growth |
|  | Gap Junction Signaling | 0.011 | 0.02 | cellular growth |
|  | FAK Signaling | 0.013 | 0.03 | cancer |
|  | Glycerolipid Metabolism | 0.013 | 0.02 | metabolism |
|  | Virus Entry via Endocytic Pathways | 0.014 | 0.03 | pathogen-influenced signaling |
|  | Interferon Signaling | 0.014 | 0.06 | immune response |
|  | Calcium Signaling | 0.014 | 0.02 | cell signaling |
|  | Acute Phase Response Signaling | 0.014 | 0.02 | immune response |
|  | Fcγ Receptor-mediated Phagocytosis in Macrophages and Monocytes | 0.015 | 0.03 | immune response |
|  | Sertoli Cell-Sertoli Cell Junction Signaling | 0.016 | 0.02 | cellular growth |
|  | IL-6 Signaling | 0.016 | 0.03 | immune response |
|  | RhoGDI Signaling | 0.016 | 0.02 | Rho GTPase pathways |
|  | Coagulation System | 0.017 | 0.05 | cardiovascular system |
|  | Nitrogen Metabolism | 0.017 | 0.02 | metabolism |
|  | NRF2-mediated Oxidative Stress Response | 0.017 | 0.02 | oxidative stress |
|  | Amyotrophic Lateral Sclerosis Signaling | 0.017 | 0.03 | disease specific |
|  | Alanine and Aspartate Metabolism | 0.018 | 0.02 | metabolism |
|  | p38 MAPK Signaling | 0.019 | 0.03 | immune response |
|  | Leukocyte Extravasation Signaling | 0.020 | 0.02 | immune response |
|  | Graft-versus-Host Disease Signaling | 0.020 | 0.04 | immune response |
|  | Role of IL-17F in Allergic Inflammatory Airway Diseases | 0.021 | 0.04 | immune response |
|  | Role of RIG1-like Receptors in Antiviral Innate Immunity | 0.022 | 0.04 | immune response |
|  | Role of Hypercytokinemia/hyperchemokinemia in the Pathogenesis of Influenza | 0.022 | 0.05 | disease specific |
|  | Role of Cytokines in Mediating Communication between Immune Cells | 0.035 | 0.04 | immune response |
|  | Hepatic Cholestasis | 0.042 | 0.02 | disease specific |
|  | T Helper Cell Differentiation | 0.048 | 0.03 | immune response |
|  |  |  |  |  |
|  |  |  |  |  |
|  |  |  |  |  |
|  |  |  |  |  |
|  |  |  |  |  |
|  | **Ingenuity Canonical Pathways** | **p-value** | **ratio** | **function** |
| **HaCaT** |  |  |  |  |
|  | Graft-versus-Host Disease Signaling | <0.001 | 0.22 | immune response |
|  | Communication between Innate and Adaptive Immune Cells | <0.001 | 0.12 | immune response |
|  | Dendritic Cell Maturation | <0.001 | 0.09 | immune response |
|  | Interferon Signaling | <0.001 | 0.22 | immune response |
|  | Antigen Presentation Pathway | <0.001 | 0.19 | immune response |
|  | Hepatic Fibrosis / Hepatic Stellate Cell Activation | <0.001 | 0.11 | disease specific |
|  | Allograft Rejection Signaling | <0.001 | 0.08 | immune response |
|  | LXR/RXR Activation | <0.001 | 0.11 | (lipid) metabolism |
|  | Role of Pattern Recognition Receptors in Recognition of Bacteria and Viruses | <0.001 | 0.13 | immune response |
|  | VDR/RXR Activation | <0.001 | 0.14 | cellular development |
|  | Cytotoxic T Lymphocyte-mediated Apoptosis of Target Cells | <0.001 | 0.09 | immune response |
|  | Autoimmune Thyroid Disease Signaling | <0.001 | 0.12 | immune response |
|  | OX40 Signaling Pathway | <0.001 | 0.08 | immune response |
|  | Activation of IRF by Cytosolic Pattern Recognition Receptors | <0.001 | 0.13 | immune response |
|  | IL-6 Signaling | <0.001 | 0.11 | immune response |
|  | IL-10 Signaling | <0.001 | 0.12 | immune response |
|  | Aryl Hydrocarbon Receptor Signaling | 0.001 | 0.08 | cell cycle/cell death |
|  | p38 MAPK Signaling | 0.001 | 0.10 | immune response |
|  | Type I Diabetes Mellitus Signaling | 0.001 | 0.09 | disease specific |
|  | Altered T Cell and B Cell Signaling in Rheumatoid Arthritis | 0.001 | 0.10 | immune response |
|  | Role of Osteoblasts, Osteoclasts and Chondrocytes in Rheumatoid Arthritis | 0.001 | 0.07 | disease specific |
|  | Cyclins and Cell Cycle Regulation | 0.001 | 0.10 | cell cycle |
|  | Atherosclerosis Signaling | 0.001 | 0.08 | disease specific |
|  | Acute Phase Response Signaling | 0.002 | 0.08 | immune response |
|  | Role of Macrophages, Fibroblasts and Endothelial Cells in Rheumatoid Arthritis | 0.002 | 0.06 | disease specific |
|  | Cell Cycle: G1/S Checkpoint Regulation | 0.002 | 0.12 | cell cycle |
|  | Cell Cycle: G2/M DNA Damage Checkpoint Regulation | 0.003 | 0.12 | cell cycle |
|  | p53 Signaling | 0.004 | 0.09 | cell cycle/cell death |
|  | T Helper Cell Differentiation | 0.005 | 0.10 | immune response |
|  | Glucocorticoid Receptor Signaling | 0.007 | 0.06 | inflammatory response |
|  | Cholecystokinin/Gastrin-mediated Signaling | 0.007 | 0.08 | nervous system |
|  | TGF-β Signaling | 0.007 | 0.09 | cellular growth |
|  | TREM1 Signaling | 0.008 | 0.09 | immune response |
|  | Crosstalk between Dendritic Cells and Natural Killer Cells | 0.008 | 0.08 | immune response |
|  | Role of Cytokines in Mediating Communication between Immune Cells | 0.008 | 0.11 | immune response |
|  | Role of IL-17A in Psoriasis | 0.009 | 0.23 | immune response |
|  | IL-17A Signaling in Gastric Cells | 0.010 | 0.16 | immune response |
|  | Eicosanoid Signaling | 0.011 | 0.08 | (lipid) metabolism |
|  | Neuroprotective Role of THOP1 in Alzheimer's Disease | 0.011 | 0.09 | disease specific |
|  | Role of IL-17F in Allergic Inflammatory Airway Diseases | 0.011 | 0.11 | immune response |
|  | PPAR Signaling | 0.012 | 0.07 | gene expression |
|  | Role of Hypercytokinemia/hyperchemokinemia in the Pathogenesis of Influenza | 0.012 | 0.11 | disease specific |
|  | IL-15 Production | 0.013 | 0.13 | immune response |
|  | β-alanine Metabolism | 0.017 | 0.05 | metabolism |
|  | JAK/Stat Signaling | 0.017 | 0.09 | cell death |
|  | Hepatic Cholestasis | 0.018 | 0.06 | disease specific |
|  | Systemic Lupus Erythematosus Signaling | 0.019 | 0.05 | disease specific |
|  | HGF Signaling | 0.020 | 0.08 | cellular growth |
|  | Cardiomyocyte Differentiation via BMP Receptors | 0.022 | 0.15 | cardiovascular system |
|  | Cdc42 Signaling | 0.023 | 0.05 | Rho GTPase pathways |
|  | Airway Pathology in Chronic Obstructive Pulmonary Disease | 0.028 | 0.22 | immune response |
|  | Pantothenate and CoA Biosynthesis | 0.030 | 0.05 | metabolism |
|  | IL-8 Signaling | 0.033 | 0.06 | immune response |
|  | IL-17 Signaling | 0.036 | 0.08 | immune response |
|  | Role of IL-17A in Arthritis | 0.036 | 0.08 | immune response |
|  | HMGB1 Signaling | 0.040 | 0.07 | immune response |
|  | NF-κB Signaling | 0.045 | 0.06 | immune response |
