# Supplementary material for: Cidofovir selectivity is based on the different response of normal and cancer cells to DNA damage
Source: BMC Med Genomics. 2013 May 23;6:18. doi: 10.1186/1755-8794-6-18 (PMC3681722; doi:10.1186/1755-8794-6-18)
Supplement: Additional file 4 — Prediction of transcription factor activities in the different cell types. The activity of the transcription factors was evaluated by P-value and regulation z-score whose calculation is based on relationships with their target genes. The relationships represent experimentally observed gene expression or transcription events associated with a direction of change that result in activation of inhibition (as derived from the literature compiled in the IPKB). The z-score predicts the identified transcription factors to be activated (positive z-score) or inhibited (negative z-score). Only upstream regulators that showed an absolute z-score > 2 in at least one of the four cell types are represented. P-values <0.05 were considered significant. [file 1755-8794-6-18-S4.docx]

**Additional file 4. Prediction of transcription factor activities in the different cell types.**

The activity of the transcription factors was evaluated by *P*-value and regulation z-score whose calculation is based on relationships with their target genes. The relationships represent experimentally observed gene expression or transcription events associated with a direction of change that result in activation of inhibition (as derived from the literature compiled in the IPKB). The z-score predicts the identified transcription factors to be activated (positive z-score) or inhibited (negative z-score). Only upstream regulators that showed an absolute z-score > 2 in at least one of the four cell types are represented. *P*-values <0.05 were considered significant.

| Upstream regulator | SiHa | | HeLa | | HaCaT | | PHKs | |
| --- | --- | --- | --- | --- | --- | --- | --- | --- |
|  | Regulation  z-score | *P*-value of  overlap | Regulation  z-score | *P*-value of  overlap | Regulation  z-score | *P*-value of  overlap | Regulation  z-score | *P*-value of  overlap |
| CDKN2A (cyclin-dependent kinase inhibitor 2A) |  |  |  |  | **2.53** | < 0.01 |  |  |
| E2f (group) ( E2F transcription factor) |  |  |  |  | **-2.44** | < 0.001 |  |  |
| EGR1 (early growth response 1) |  |  |  |  | **2.49** | < 0.001 |  |  |
| ESR1 (estrogen receptor 1) |  |  |  |  |  |  | **-2.10** | < 0.01 |
| FOSL1 (FOS-like antigen 1) |  |  |  |  | **2.03** | < 0.001 |  |  |
| FOXA2 (forkhead box A2) |  |  |  |  |  |  | **-2.24** | 0.417 |
| FOXL2 (forkhead box L2) |  |  |  |  | **2.21** | < 0.001 |  |  |
| GLI1 (GLI family zinc finger 1) |  |  |  |  | **2.22** | < 0.001 |  |  |
| HMGB1 (high mobility group box 1) |  |  |  |  | **2.25** | < 0.001 |  |  |
| IFI16 (interferon, gamma-inducible protein 16) |  |  |  |  | **2.06** | < 0.001 |  |  |
| IRF3 (interferon regulatory factor 3) |  |  |  |  | **2.72** | < 0.001 |  |  |
| IRF7 (interferon regulatory factor 7) |  |  |  |  | **3.49** | < 0.001 |  |  |
| KDM5B [lysine (K)-specific demethylase 5B] |  |  |  |  | **2.20** | < 0.001 |  |  |
| MYC [v-myc myelocytomatosis viral oncogene homolog (avian)] |  |  | **-2.03** | < 0.01 | **-3.75** | < 0.001 |  |  |
| MYCN [v-myc myelocytomatosis viral related oncogene, neuroblastoma derived (avian)] | **-2.23** | 0.053 | **-2.34** | < 0.05 | **-3.52** | 0.078 |  |  |
| Nfat (family) (nuclear factor of activated T-cells) |  |  |  |  | **2.18** | < 0.001 |  |  |
| NFkB (complex) (nuclear factor kappa) |  |  |  |  | **2.72** | < 0.001 |  |  |
| NR1H3 (nuclear receptor subfamily 1, group H, member 3) |  |  |  |  |  |  | **-2.45** | 0.534 |
| NR3C1 [nuclear receptor subfamily 3, group C, member 1 (glucocorticoid receptor)] |  |  |  |  | **-2.07** | < 0.001 |  |  |
| REL [v-rel reticuloendotheliosis viral oncogene homolog (avian)] |  |  |  |  | **2.15** | < 0.001 |  |  |
| SMARCA4 (SWI/SNF related, matrix associated, actin dependent regulator of chromatin, subfamily a, member 4) |  |  |  |  | **2.53** | < 0.001 |  |  |
| SMARCB1 (SWI/SNF related, matrix associated, actin dependent regulator of chromatin, subfamily b, member 1) |  |  |  |  | **2.54** | <0.05 | **-2.89** | < 0.001 |
| SNAI1 [snail homolog 1 (Drosophila)] |  |  |  |  |  |  | **2.06** | < 0.001 |
| STAT2 (signal transducer and activator of transcription 2) |  |  |  |  | **2.34** | < 0.001 |  |  |
| TBX2 (T-box 2) |  |  |  |  | **-2.64** | < 0.05 | **2.47** | <0.001 |
| TCF3 [transcription factor 3 (E2A immunoglobulin enhancer binding factors E12/E47)] |  |  |  |  |  |  | **-2.27** | < 0.001 |
| THRB (thyroid hormone receptor, beta) |  |  |  |  | **-2.19** | < 0.05 |  |  |
| TP53 (tumor protein p53) |  |  | **2.07** | < 0.001 | **2.71** | < 0.001 |  |  |
| VDR [vitamin D (1,25- dihydroxyvitamin D3) receptor] |  |  |  |  |  |  | **2.02** | < 0.05 |
