# Supplementary material for: Cidofovir selectivity is based on the different response of normal and cancer cells to DNA damage
Source: BMC Med Genomics. 2013 May 23;6:18. doi: 10.1186/1755-8794-6-18 (PMC3681722; doi:10.1186/1755-8794-6-18)
Supplement: Additional file 5 — Inflammatory response networks. Networks were constructed with IPA software using genes DE and involved in ‘inflammatory response’ following CDV treatment of (A) SiHa, (B) HeLa, (C) HaCaT, or (D) PHKs. [file 1755-8794-6-18-S5.pdf]

Additional file 5. Inflammatory response networks.

Networks were constructed with IPA software using genes DE and involved in 'inflammatory response' following CDV treatment of (A) SiHa, (B) HeLa, (C) HaCaT, or (D) PHKs with associated pathways. Upregulated genes in red, downregulated genes in green.

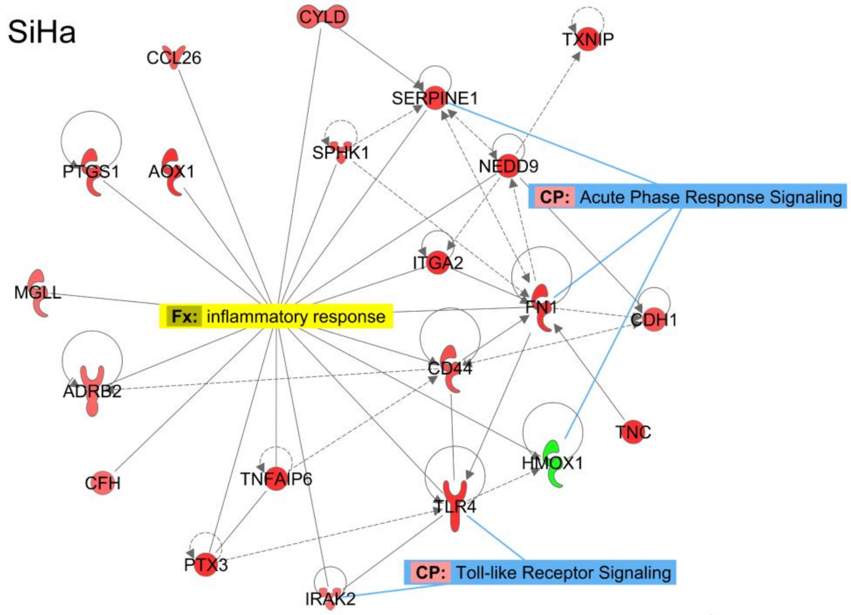

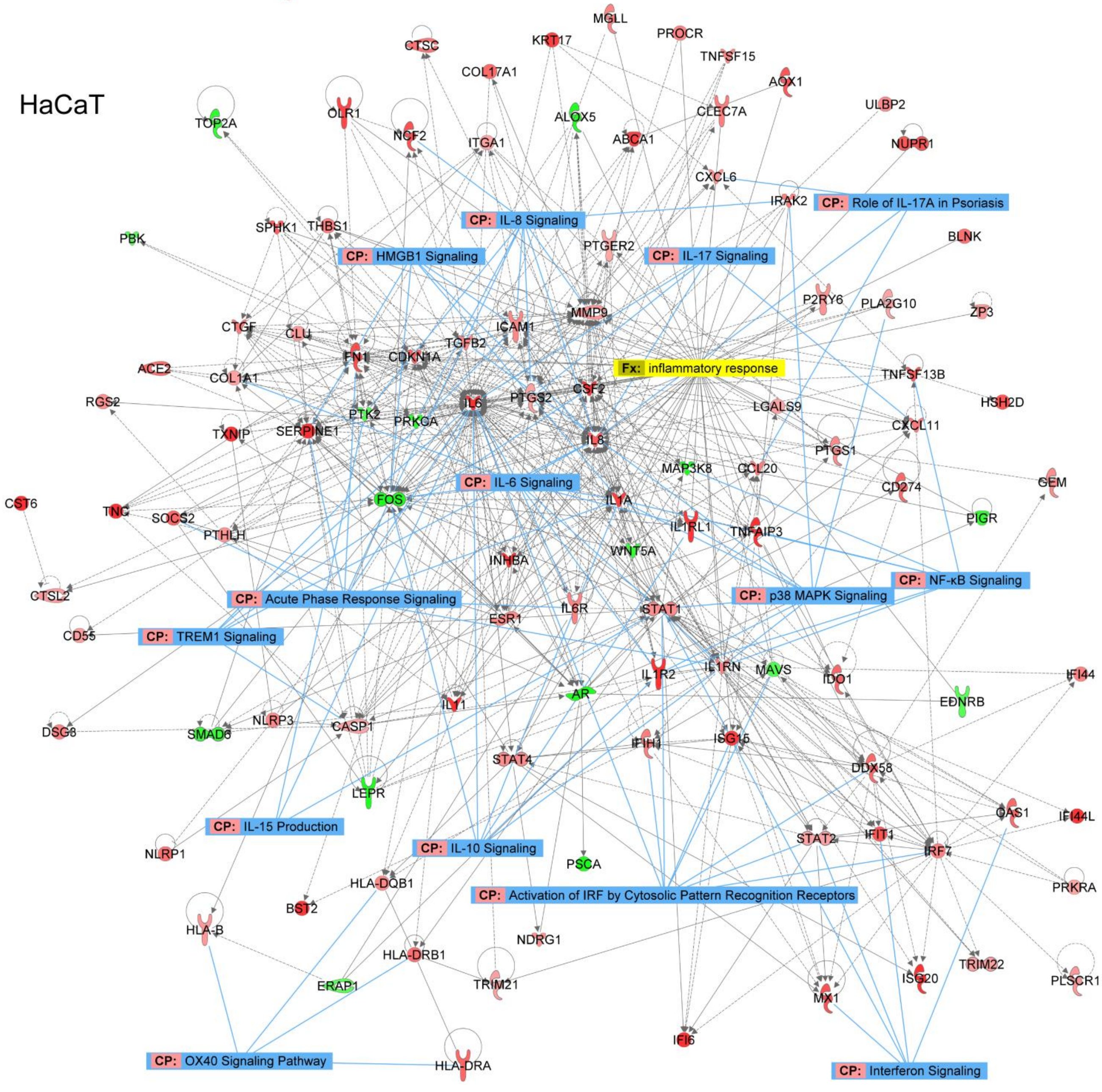

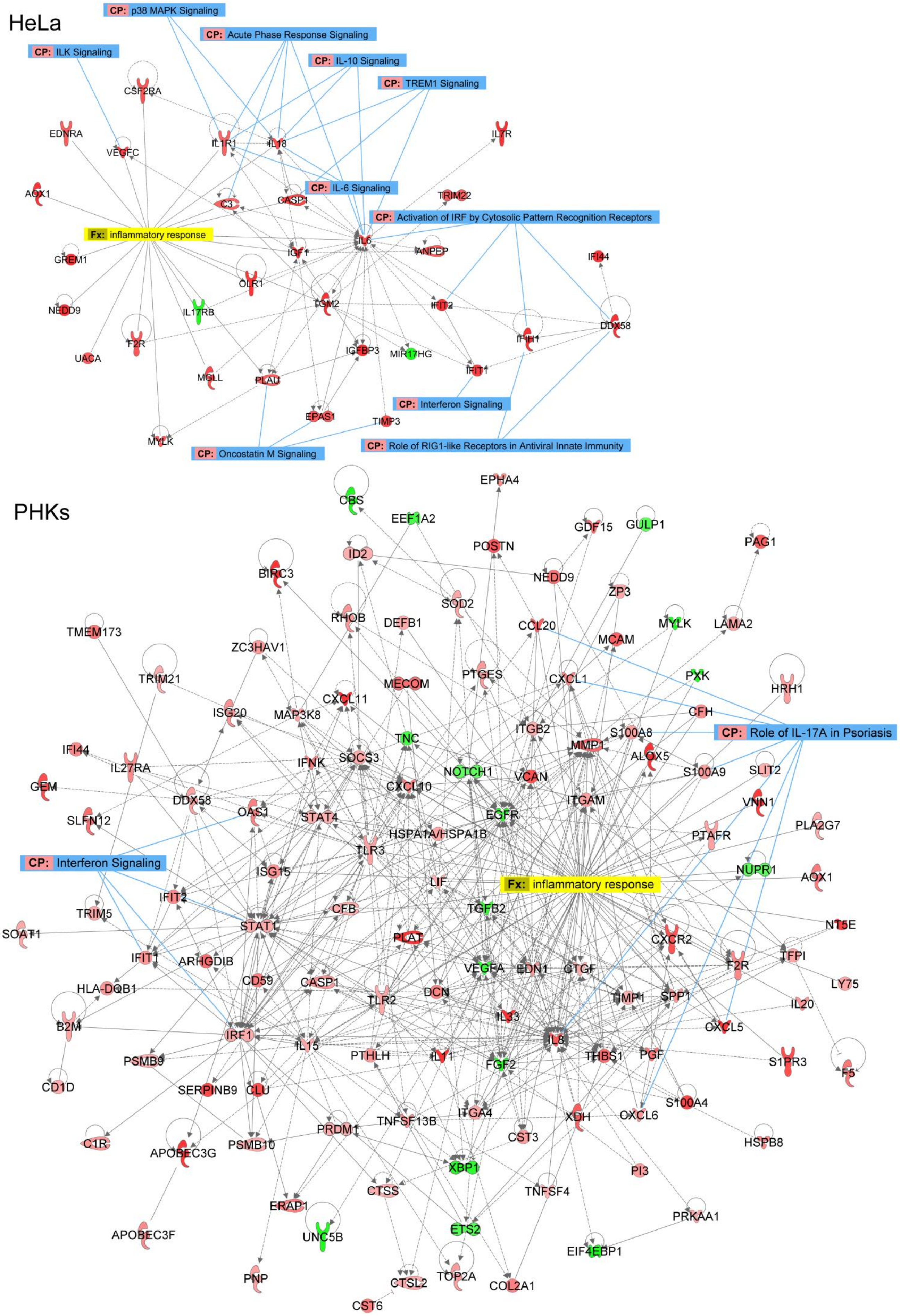
HeLa
CP: p38 MAPK Signaling
CP: Acute Phase Response Signaling
CP: ILK Signaling
CP: IL-10 Signaling
CP: TREM1 Signaling
CP: IL-6 Signaling
CP: Activation of IRF by Cytosolic Pattern Recognition Receptors
Fx: inflammatory response
CP: Interferon Signaling
CP: Oncostatin M Signaling
CP: Role of RIG1-like Receptors in Antiviral Innate Immunity
CSF2RA
EDNRA
VEGFC
IL1R1
IL18
IL7R
AOX1
C3
CASP1
TRIM22
IL6
GREM1
IGF1
ANPEP
IFI44
OLR1
NEDD9
IL17RB
TGM2
IFIT2
DDX58
IFIH1
F2R
UACA
IGFBP3
MIR17HG
IFIT1
MGLL
PLAU
EPAS1
TIMP3
MYLK
PHKs
CBS
EPHA4
EEF1A2
GDF15
GULP1
ID2
POSTN
PAG1
BIRC3
NEDD9
ZP3
SOD2
RHOB
DEFB1
CCL20
MYLK
LAMA2
TMEM173
MCAM
ZC3HAV1
TRIM21
PTGES
MECOM
CXCL1
PXK
HRH1
CXCL11
ISG20
MAP3K8
CFH
ITGB2
S100A8
CP: Role of IL-17A in Psoriasis
TNC
IFI44
SOCS3
MMP1
IL27RA
IFNK
ALOX5
NOTCH1
VCAN
S100A9
SLIT2
GEM
CXCL10
DDX58
ITGAM
VNN1
OAS1
STAT4
EGFR
SLFN12
PLA2G7
HSPA1A/HSPA1B
PTAFR
TLR3
CP: Interferon Signaling
ISG15
NUPR1
AOX1
LIF
Fx: inflammatory response
IFIT2
TRIM5
CFB
TGFB2
STAT1
NT5E
SOAT1
PLAT
CXCR2
IFIT1
ARHGDIB
F2R
TFPI
VEGFA
EDN1
CTGF
CD59
CASP1
HLA-DQB1
LY75
DCN
TIMP1
SPP1
TLR2
IL20
B2M
IL33
CXCL5
IRF1
IL8
IL15
PSMB9
PTHLH
IL1
THBS1
PGF
S1PR3
FGF2
F5
CD1D
SERPINB9
CLU
S100A4
ITGA4
XDH
CXCL6
TNFSF13B
PRDM1
C1R
CST3
HSPB8
APOBEC3G
PSMB10
XBP1
PI3
CTSS
TNFSF4
ERAP1
PRKAA1
UNC5B
ETS2
APOBEC3F
EIF4EBP1
PNP
CTSL2
TOP2A
COL2A1
CST6
