# Supplementary material for: Cidofovir selectivity is based on the different response of normal and cancer cells to DNA damage
Source: BMC Med Genomics. 2013 May 23;6:18. doi: 10.1186/1755-8794-6-18 (PMC3681722; doi:10.1186/1755-8794-6-18)
Supplement: Additional file 6 — Effect of CDV on ‘cell cycle’ and ‘DNA replication, recombination, and repair’ in HPV- cells. Genes modulated by CDV in HaCaT and/or PHKs that are involved in pathways related to ‘cell cycle’ and ‘DNA replication, recombination, and repair’. [file 1755-8794-6-18-S6.docx]

**Additional file 6. Effect of CDV on ‘cell cycle’ and ‘DNA replication, recombination, and repair’ in HPV^-^ cells.**

Genes modulated by CDV in HaCaT and/or PHKs are involved in pathways related to ‘cell cycle’ and ‘DNA replication, recombination, and repair’

**Genes similarly modulated by CDV in HaCaT and PHKs that are involved in pathways related to ‘cell cycle’ and ‘DNA replication, recombination, and repair’**

| Gene | Full name | Protein function | Differential expression | |
| --- | --- | --- | --- | --- |
|  |  |  | **HaCaT** | **PHKs** |
| *CYP1B1* | cytochrome P450, family 1, subfamily B, polypeptide 1 | member of the cytochrome P450 superfamily of enzymes; the cytochrome P450 proteins are monooxygenases which catalyze many reactions involved in drug metabolism and synthesis of cholesterol, steroids and other lipids | **↓** | **↓** |
| *THBS1* | thrombospondin 1 | subunit of a disulfide-linked homotrimeric protein; adhesive glycoprotein that mediates cell-to-cell and cell-to-matrix interactions | **↑** | **↑** |

**Genes oppositely modulated by CDV in HaCaT and PHKs that are involved in pathways related to ‘cell cycle’ and ‘DNA replication, recombination, and repair’**

| Gene | Full name | Protein function | Differential expression | |
| --- | --- | --- | --- | --- |
|  |  |  | **HaCaT** | **PHKs** |
| *ABCG2* | ATP-binding cassette, sub-family G (WHITE), member 2 | member of the superfamily of ATP-binding cassette (ABC) transporters that transport various molecules across extra- and intra-cellular membranes; also knwon as breast cancer resistance protein because it functions as a xenobiotic transporter playing a major role in multi-drug resistance | **↓** | **↑** |
| *ALOX5* | arachidonate 5-lipoxygenase | catalyzes the first step in leukotriene biosynthesis, and thereby plays a role in inflammatory processes | **↓** | **↑** |
| *BUB1* | budding uninhibited by benzimidazoles 1 homolog (yeast), mitotic spindle checkpoint kinase | kinase involved in spindle checkpoint function; functions in part by phosphorylating a member of the miotic checkpoint complex and activating the spindle checkpoint | **↓** | **↑** |
| *CCNA2* | cyclin A2 | essential for the control of the cell cycle at the G1/S (start) and the G2/M (mitosis) transitions | **↓** | **↑** |
| *CCNB1* | cyclin B1 | essential for the control of the cell cycle at the G2/M (mitosis) transition; complexes with p34(cdc2) to form the maturation-promoting factor (MPF) | **↓** | **↑** |
| *DEPDC1* | DEP domain containing 1 | unknown | **↓** | **↑** |
| *DLGAP5* | discs, large (Drosophila) homolog-associated protein 5 | potential cell cycle regulator that may play a role in carcinogenesis of cancer cells; mitotic phosphoprotein regulated by the ubiquitin-proteasome pathway; key regulator of adherens junction integrity and differentiation that may be involved in CDH1-mediated adhesion and signaling in epithelial cells | **↓** | **↑** |
| *MAP3K8* | mitogen-activated protein kinase kinase kinase 8 | member of the serine/threonine protein kinase family; localizes to the cytoplasm and can activate both the MAP kinase and JNK kinase pathways; was shown to activate IkappaB kinases, and thus induce the nuclear production of NF-kappaB | **↓** | **↑** |
| *MKI67* | antigen identified by monoclonal antibody Ki-67 | nuclear protein that is associated with and may be necessary for cellular proliferation | **↓** | **↑** |
| *NUF2* | NDC80 kinetochore complex component, homolog (S. cerevisiae) | component of the essential kinetochore- associated NDC80 complex (including Ndc80, Nuf2, Spc24, and Spc25) , which is required for chromosome segregation and spindle checkpoint activity | **↓** | **↑** |
| *PBK* | PDZ binding kinase | phosphorylates MAP kinase p38. Seems to be active only in mitosis; may also play a role in the activation of lymphoid cells. When phosphorylated, forms a complex with TP53, leading to TP53 destabilization and attenuation of G2/M checkpoint during doxorubicin-induced DNA damage | **↓** | **↑** |
| *SPC25* | NDC80 kinetochore complex component, homolog (S. cerevisiae) | component of the essential kinetochore- associated NDC80 complex, which is required for chromosome segregation and spindle checkpoint activity | **↓** | **↑** |
| *SSPN* | sarcospan (Kras oncogene-associated gene) | member of the dystrophin-glycoprotein complex (DGC) that forms a link between the F-actin cytoskeleton and the extracellular matrix | **↓** | **↑** |
| *TOP2A* | topoisomerase (DNA) II alpha 170kDa | controls and alters the topologic states of DNA during transcription | **↓** | **↑** |
| *NUPR1* | nuclear protein, transcriptional regulator, 1 | is induced by several stresses; regulates transcription, cell cycle, apoptosis, autophagy, chromatin accessibility and its functions depend on its molecular partners, its cellular location, the cell-types concerned and its expression level | **↑** | **↓** |
| *Nrg1* | neuregulin 1 | interacts with the NEU/ERBB2 receptor tyrosine kinase to increase its phosphorylation on tyrosine residues; is a signaling protein that mediates cell-cell interactions and plays critical roles in the growth and development of multiple organ systems | **↑** | **↓** |
| *TGFB2* | transforming growth factor, beta 2 | member of the TGFB family of cytokines, which regulate proliferation, differentiation, adhesion, migration, and other functions in many cell types by transducing their signal through combinations of transmembrane type I and type II receptors (TGFBR1 and TGFBR2) and their downstream effectors, the SMAD proteins | **↑** | **↓** |
| *TNC* | tenascin C | extracellular matrix protein with a spatially and temporally restricted tissue distribution | **↑** | **↓** |
| *TP63* | tumor protein p63 | member of the p53 family of transcription factors; encodes two groups of protein isoforms: TA-isoforms that function as tumor suppressors and ∆N-isoforms as oncoproteins; it was reported that treatment with DNA damage agents down-regulates ∆Np63α at the transcription level inducing apoptosis in HaCaT cells that carry mutant p53 | **↑** | **↓** |

**Genes exclusively modulated by CDV in HaCaT that are involved in pathways related to ‘cell cycle’ and ‘DNA replication, recombination, and repair’**

| Gene | Full name | Protein function | Differential expression |
| --- | --- | --- | --- |
| *AKT3* | v-akt murine thymoma viral oncogene homolog 3 (protein kinase B, gamma) | member of the AKT, also called PKB, serine/threonine protein kinase family. AKT kinases are regulators of cell signaling in response to insulin and growth factors; they are involved in a wide variety of biological processes including cell proliferation, differentiation, apoptosis, tumorigenesis, as well as glycogen synthesis and glucose uptake. This kinase has been shown to be stimulated by platelet-derived growth factor (PDGF), insulin, and insulin-like growth factor 1 (IGF1) and may play a role in regulating cell survival | **↓** |
| *ALDH3A2* | aldehyde dehydrogenase 3 family, member A2 | plays a major role in the detoxification of aldehydes generated by alcohol metabolism and lipid peroxidation; catalyzes the oxidation of long-chain aliphatic aldehydes to fatty acid. | **↓** |
| *ALDH6A1* | aldehyde dehydrogenase 6 family, member A1 | mitochondrial methylmalonate semialdehyde dehydrogenase that plays a role in the valine and pyrimidine catabolic pathways; catalyzes the irreversible oxidative decarboxylation of malonate and methylmalonate semialdehydes to acetyl- and propionyl-CoA | **↓** |
| *CDC25C* | cell division cycle 25 homolog C (S. pombe) | tyrosine protein phosphatase that belongs to the Cdc25 phosphatase family; is required for progression of the cell cycle; directs dephosphorylation of cyclin B-bound CDC2 and triggers entry into mitosis | **↓** |
| *FOS* | FBJ murine osteosarcoma viral oncogene homolog | one of the four members of the FOS family that includes FOS, FOSB, FOSL1, and FOSL2; they are leucine zipper proteins that can dimerize with proteins of the JUN family, thereby forming the transcription factor complex AP-1; as such, the FOS proteins have been implicated as regulators of cell proliferation, differentiation, and transformation | **↓** |
| *HDAC8* | histone deacetylase 8 | belongs to class I of the histone deacetylase family; catalyzes the deacetylation of lysine residues in the histone N-terminal tails and represses transcription in large multiprotein complexes with transcriptional co-repressors; histones play a critical role in transcriptional regulation, cell cycle progression, and developmental events and histone acetylation/deacetylation alters chromosome structure and affects transcription factor access to DNA | **↓** |
| *KIF23* | kinesin family member 23 | member of kinesin-like protein family that includes microtubule-dependent molecular motors that transport organelles within cells and move chromosomes during cell division; cross-bridges antiparallel microtubules and drives microtubule movement in vitro | **↓** |
| *MCM6* | minichromosome maintenance complex component 6 | is one of the highly conserved mini-chromosome maintenance proteins (MCM) that are essential for the initiation of eukaryotic genome replication; the hexameric protein complex formed by the MCM proteins is a key component of the pre-replication complex (pre_RC) and is involved in the formation of replication forks and in the recruitment of other DNA replication related proteins; the MCM complex consisting of this protein and MCM2, 4 and 7 proteins possesses DNA helicase activity, and may act as a DNA unwinding enzyme | **↓** |
| *NFIB* | nuclear factor I/B | member of the NF1 (nuclear factor 1) family of transcription factors; recognizes and binds the palindromic sequence 5'- TTGGCNNNNNGCCAA-3' present in viral and cellular promoters; nuclear factors are individually capable of activating transcription and replication | **↓** |
| *NR2F1* | nuclear receptor subfamily 2, group F, member 1 | transcription factor that binds to the ovalbumin promoter and, in conjunction with another protein (S300-II) stimulates initiation of transcription; binds to both direct repeats and palindromes of the 5'-AGGTCA-3' motif | **↓** |
| *PPM1L* | protein phosphatase, Mg2+/Mn2+ dependent, 1L | acts as a suppressor of the SAPK signaling pathways by associating with and dephosphorylating MAP3K7/TAK1 and MAP3K5, and by attenuating the association between MAP3K7/TAK1 and MAP2K4 or MAP2K6 | **↓** |
| *SERPINB5* | serpin peptidase inhibitor, clade B (ovalbumin), member 5 | tumor suppressor; blocks the growth, invasion, and metastatic properties of mammary tumors; as it does not undergo the S (stressed) to R (relaxed) conformational transition characteristic of active serpins, it exhibits no serine protease inhibitory activity | **↓** |
| *SKP2* | S-phase kinase-associated protein 2 (p45), E3 ubiquitin protein ligase | member of the F-box protein family; substrate recognition component of a SCF (SKP1-CUL1-F- box protein) E3 ubiquitin-protein ligase complex which mediates the ubiquitination and subsequent proteasomal degradation of target proteins involved in cell cycle progression, signal transduction and transcription; essential element of the cyclin A-CDK2 S-phase kinase; specifically recognizes phosphorylated cyclin-dependent kinase inhibitor 1B (CDKN1B, also referred to as p27 or KIP1) predominantly in S phase and interacts with S-phase kinase-associated protein 1 (SKP1 or p19) | **↓** |
| *CDKN1A* | cyclin-dependent kinase inhibitor 1A (p21, Cip1) | binds to and inhibits the activity of cyclin-CDK2 or -CDK4 complexes, and thus functions as a regulator of cell cycle progression at G1; gene expression tightly controlled by the tumor suppressor protein p53, through which p21 mediates the p53-dependent cell cycle G1 phase arrest in response to a variety of stress stimuli | **↑** |
| *CCND2* | cyclin D2 | forms a complex with and functions as a regulatory subunit of CDK4 or CDK6, whose activity is required for cell cycle G1/S transition; interacts with and is involved in the phosphorylation of tumor suppressor protein Rb | **↑** |
| *ESR1* | estrogen receptor 1 | estrogen receptor, a ligand-activated transcription factor composed of several domains important for hormone binding, DNA binding, and activation of transcription; localizes to the nucleus where it may form a homodimer or a heterodimer with estrogen receptor 2 | **↑** |
| *GADD45A* | growth arrest and DNA-damage-inducible, alpha | its transcript levels are increased following stressful growth arrest conditions and treatment with DNA-damaging agents; responds to environmental stresses by mediating activation of the p38/JNK pathway via MTK1/MEKK4 kinase; the DNA damage-induced transcription of this gene is mediated by both p53-dependent and -independent mechanisms | **↑** |
| *IL1A* | interleukin 1, alpha | pleiotropic cytokine involved in various immune responses, inflammatory processes, and hematopoiesis | **↑** |
| *IL6* | interleukin 6 | is implicated in a wide variety of inflammation-associated disease states | **↑** |
| *SERPINE2* | serpin peptidase inhibitor, clade E (nexin, plasminogen activator inhibitor type 1), member 2 | member of the serpin family of proteins, a group of proteins that inhibit serine proteases; inhibits the proteases thrombin, urokinase, plasmin and trypsin. | **↑** |
| *SNAI2* | snail homolog 2 (Drosophila) | transcriptional repressor; involved in epithelial-mesenchymal transition and invasion | **↑** |

**Genes exclusively modulated by CDV in PHKs that are involved in pathways related to ‘cell cycle’ and ‘DNA replication, recombination, and repair’**

| Gene | Full name | Protein function | Differential expression |
| --- | --- | --- | --- |
| *ALDH1L1* | aldehyde dehydrogenase 1 family, member L1 | catalyzes the conversion of 10-formyltetrahydrofolate, nicotinamide adenine dinucleotide phosphate (NADP+), and water to tetrahydrofolate, NADPH, and carbon dioxide; belongs to the aldehyde dehydrogenase family; loss of function or expression of this gene is associated with decreased apoptosis, increased cell motility, and cancer progression. | **↓** |
| *CDK6* | cyclin-dependent kinase 6 | catalytic subunit of the protein kinase complex that is important for cell cycle G1 phase progression and G1/S transition; it activity first appears in mid-G1 phase, which is controlled by the regulatory subunits including D-type cyclins and members of INK4 family of CDK inhibitors; this kinase, as well as CDK4, has been shown to phosphorylate, and thus regulate the activity of, tumor suppressor protein Rb | **↓** |
| *CYP1A1* | cytochrome P450, family 1, subfamily A, polypeptide 1 | member of the cytochrome P450 superfamily of enzymes that are monooxygenases which catalyze many reactions involved in drug metabolism and synthesis of cholesterol, steroids and other lipids | **↓** |
| *NFIA* | nuclear factor I/A | member of the NF1 (nuclear factor 1) family of transcription factors; recognizes and binds the palindromic sequence 5'- TTGGCNNNNNGCCAA-3' present in viral and cellular promoters; nuclear factors are individually capable of activating transcription and replication | **↓** |
| *NCOA7* | nuclear receptor coactivator 7 | Enhances the transcriptional activities of several nuclear receptors. Involved in the coactivation of different nuclear receptors, such as ESR1, THRB, PPARG and RARA | **↓** |
| *STAG1* | stromal antigen 1 | component of cohesin complex, a complex required for the cohesion of sister chromatids after DNA replication; the cohesin complex apparently forms a large proteinaceous ring within which sister chromatids can be trapped; at anaphase, the complex is cleaved and dissociates from chromatin, allowing sister chromatids to segregate; the cohesin complex may also play a role in spindle pole assembly during mitosis | **↓** |
| *ALDH1A1* | aldehyde dehydrogenase 1 family, member A1 | binds free retinal and cellular retinol-binding protein- bound retinal; can convert/oxidize retinaldehyde to retinoic acid | **↑** |
| *ALDH7A1* | aldehyde dehydrogenase 7 family, member A1 | member of subfamily 7 in the aldehyde dehydrogenase gene family that play a major role in the detoxification of aldehydes generated by alcohol metabolism and lipid peroxidation | **↑** |
| *ANAPC7* | anaphase promoting complex subunit 7 | tetratricopeptide repeat containing component of the anaphase promoting complex/cyclosome (APC/C), a large E3 ubiquitin ligase that controls cell cycle progression by targeting a number of cell cycle regulators such as B-type cyclins for 26S proteasome-mediated degradation through ubiquitination; is required for proper protein ubiquitination function of APC/C and for the interaction of APC/C with certain transcription coactivators | **↑** |
| *BIRC5* | baculoviral IAP repeat-containing 5 (apoptosis inhibitor surviving) | member of the inhibitor of apoptosis (IAP) gene family, which encode negative regulatory proteins that prevent apoptotic cell death; IAP family members usually contain multiple baculovirus IAP repeat (BIR) domains, but BIRC5 has only a single BIR domain; is a component of the chromosomal passenger complex (CPC), a complex that acts as a key regulator of mitosis; the CPC complex has essential functions at the centromere in ensuring correct chromosome alignment and segregation and is required for chromatin-induced microtubule stabilization and spindle assembly. | **↑** |
| *BRCA1* | breast cancer 1, early onset | nuclear phosphoprotein that plays a role in maintaining genomic stability, and it also acts as a tumor suppressor; the BRCA1-BARD1 heterodimer coordinates a diverse range of cellular pathways (such as DNA damage repair, ubiquitination and transcriptional regulation to maintain genomic stability); acts by mediating ubiquitin E3 ligase activity that is required for its tumor suppressor function; plays a central role in DNA repair by facilitating cellular response to DNA repair; required for appropriate cell cycle arrests after ionizing irradiation in both the S-phase and the G2 phase of the cell cycle; involved in transcriptional regulation of P21 in response to DNA damage | **↑** |
| *CCNE2* | cyclin E2 | forms a complex with and functions as a regulatory subunit of CDK2; specifically interacts with CIP/KIP family of CDK inhibitors, and plays a role in cell cycle G1/S transition; its expression peaks at the G1-S phase and exhibits a pattern of tissue specificity distinct from that of cyclin E1 | **↑** |
| *CDC23* | cell division cycle 23 homolog (S. cerevisiae) | essential for cell cycle progression through the G2/M transition; ; is a component of anaphase-promoting complex (APC), which is composed of eight protein subunits and highly conserved in eukaryotic cells. APC catalyzes the formation of cyclin B-ubiquitin conjugate that is responsible for the ubiquitin-mediated proteolysis of B-type cyclins. This protein and 3 other members of the APC complex contain the TPR (tetratricopeptide repeat), a protein domain important for protein-protein interaction | **↑** |
| *CDC25A* | cell division cycle 25 homolog A (S. pombe) | member of the CDC25 family of tyrosine protein phosphatases; is required for progression from G1 to the S phase of the cell cycle; activates the cyclin-dependent kinase CDC2 by removing two phosphate groups; is specifically degraded in response to DNA damage, which prevents cells with chromosomal abnormalities from progressing through cell division | **↑** |
| *CDC6* | cell division cycle 6 homolog (S. cerevisiae) | involved in the initiation of DNA replication; participates in checkpoint controls that ensure DNA replication is completed before mitosis is initiated | **↑** |
| *CDC7* | cell division cycle 7 homolog (S. cerevisiae) | has protein kinase activity; phosphorylates critical substrates that regulate the G1/S phase transition and/or DNA replication; can phosphorylates MCM2 and MCM3 | **↑** |
| *CDK1* | cyclin-dependent kinase 1; CDC2 | member of the Ser/Thr protein kinase family ; plays a key role in the control of the eukaryotic cell cycle; required in higher cells for entry into S-phase and mitosis; is a catalytic subunit of the highly conserved protein kinase complex known as M-phase promoting factor (MPF), which is essential for G1/S and G2/M phase transitions of eukaryotic cell cycle; mitotic cyclins stably associate with this protein and function as regulatory subunits; its kinase activity is controlled by cyclin accumulation and destruction through the cell cycle; its phosphorylation and its dephosphorylation also play important regulatory roles in cell cycle control | **↑** |
| *CDT1* | chromatin licensing and DNA replication factor 1 | involved in the formation of the pre-replication complex that is necessary for DNA replication; can bind geminin, which prevents replication and may function to prevent this protein from initiating replication at inappropriate origins; phosphorylation of this protein by cyclin A-dependent kinases results in degradation of the protein | **↑** |
| *CKS2* | CDC28 protein kinase regulatory subunit 2 | binds to the catalytic subunit of the cyclin dependent kinases and is essential for their biological function. | **↑** |
| *CTNNB1* | catenin (cadherin-associated protein), beta 1, 88kDa | is part of a complex of proteins that constitute adherens junctions (AJs); involved in the regulation of cell adhesion and in signal transduction through the Wnt pathway | **↑** |
| *DHFR* | dihydrofolate reductase | converts dihydrofolate into tetrahydrofolate, a methyl group shuttle required for the de novo synthesis of purines, thymidylic acid, and certain amino acids. | **↑** |
| *DRAM1* | DNA-damage regulated autophagy modulator 1 | lysosomal modulator of autophagy that plays a central role in p53/TP53-mediated apoptosis | **↑** |
| *GEN1* | Gen homolog 1, endonuclease (Drosophila) | endonuclease which cleaves flap structures at the junction between single-stranded DNA and double-stranded DNA | **↑** |
| *GSTT2* | glutathione S-transferase theta 2 | member of the theta class of the superfamily of glutathione S-transferase proteins that catalyze the conjugation of reduced glutathione to a variety of electrophilic and hydrophobic compounds | **↑** |
| *GSTM3* | glutathione S-transferase mu 3 (brain) | belongs to the mu class of glutathione S-transferases; functions in the detoxification of electrophilic compounds, including carcinogens, therapeutic drugs, environmental toxins and products of oxidative stress, by conjugation with glutathione | **↑** |
| *KAT2B* | K(lysine) acetyltransferase 2B | associates with p300/CBP, being CBP and p300 large nuclear proteins that bind to many sequence-specific factors involved in cell growth and/or differentiation | **↑** |
| *KIF11* | kinesin family member 11 | motor protein that belongs to the kinesin-like protein family; involved in various kinds of spindle dynamics; functions in chromosome positioning, centrosome separation and establishing a bipolar spindle during cell mitosis | **↑** |
| *MDM2* | Mdm2 p53 binding protein homolog (mouse) | nuclear phosphoprotein that binds and inhibits transactivation by tumor protein p53, as part of an autoregulatory negative feedback loop; its transcription is modulated by the transcription factor tumor protein p53; has E3 ubiquitin ligase activity, which targets tumor protein p53 for proteasomal degradation; also affects the cell cycle, apoptosis, and tumorigenesis through interactions with other proteins, including retinoblastoma 1 and ribosomal protein L5; MDM2 overexpression can result in excessive inactivation of tumor protein p53, diminishing its tumor suppressor function | **↑** |
| *NBN* | nibrin | member of the MRE11/RAD50 double-strand break repair complex which consists of 5 proteins; is involved in DNA double-strand break repair and DNA damage-induced checkpoint activation | **↑** |
| *ORC6* | origin recognition complex subunit 6 | is a subunit of the origin recognition complex (ORC), a highly conserved six subunit protein complex essential for the initiation of the DNA replication in eukaryotic cells; ORC binds specifically to origins of replication and serves as a platform for the assembly of additional initiation factors | **↑** |
| *PCNA* | proliferating cell nuclear antigen | is an auxiliary protein of DNA polymerase delta and is involved in the control of eukaryotic DNA replication by increasing the polymerase's processibility during elongation of the leading strand; in response to DNA damage, this protein is ubiquitinated and is involved in the RAD6-dependent DNA repair pathway | **↑** |
| *PIDD* | p53-induced death domain protein | contains a leucine-rich repeat and a death domain; interacts with other death domain proteins, such as Fas (TNFRSF6)-associated via death domain (FADD) and MAP-kinase activating death domain-containing protein (MADD), and thus may function as an adaptor protein in cell death-related signaling processes | **↑** |
| *PLAGL1* | pleiomorphic adenoma gene-like 1 | C2H2 zinc finger protein with transactivation and DNA-binding activities; has been shown to have anti-proliferative properties, and thus thought to function as a tumor suppressor | **↑** |
| *PMAIP1* | phorbol-12-myristate-13-acetate-induced protein 1 | promotes activation of caspases and apoptosis; promotes mitochondrial membrane changes and efflux of apoptogenic proteins from the mitochondria; contributes to p53-dependent apoptosis after radiation exposure | **↑** |
| *RAD51* | RAD51 homolog (RecA homolog, E. coli) (S. cerevisiae) | member of the RAD51 protein family which are involved in the homologous recombination and repair of DNA; interacts with the ssDNA-binding protein RPA and RAD52, and plays roles in homologous recombination and double-strand break repair; binds to single and double stranded DNA and exhibits DNA-dependent ATPase activity; interacts with BRCA1 and BRCA2, which may be important for the cellular response to DNA damage; BRCA2 regulates both the intracellular localization and DNA-binding ability of this protein | **↑** |
| *RFC3* | replication factor C (activator 1) 3 | is an auxiliary protein of DNA polymerase delta and epsilon; the elongation of primed DNA templates by DNA polymerase delta and epsilon requires the action of the accessory proteins proliferating cell nuclear antigen (PCNA) and activator 1 | **↑** |
| *RRM2B* | ribonucleotide reductase M2 B (TP53 inducible) | small subunit of a p53-inducible ribonucleotide reductase; this heterotetrameric enzyme catalyzes the conversion of ribonucleoside diphosphates to deoxyribonucleoside diphosphates and the product of this reaction is necessary for DNA synthesis | **↑** |
| *TP53AIP1* | tumor protein p53 regulated apoptosis inducing protein 1 | may play an important role in mediating p53/TP53- dependent apoptosis | **↑** |
| *TP53INP1* | tumor protein p53 inducible nuclear protein 1 | in response to double-strand DNA breaks, promotes p53/TP53 phosphorylation on 'Ser-46' and subsequent apoptosis | **↑** |
| *TP53I3* | tumor protein p53 inducible protein 3 | is similar to oxidoreductases, which are enzymes involved in cellular responses to oxidative stresses and irradiation; its transcription is induced by the tumor suppressor p53 and is thought to be involved in p53-mediated cell death | **↑** |
